# Integrating epidemiological and genetic data with different sampling intensities into a dynamic model of respiratory syncytial virus transmission

**DOI:** 10.1038/s41598-021-81078-x

**Published:** 2021-01-14

**Authors:** Ivy K. Kombe, Charles N. Agoti, Patrick K. Munywoki, Marc Baguelin, D. James Nokes, Graham F. Medley

**Affiliations:** 1 KEMRI-Wellcome Trust Research Programme, KEMRI Centre for Geographical Medical Research-Coast, P.O. Box 230-80108, Kilifi, Kenya; 2 Centre for Mathematical Modelling of Infectious Disease and Department of Global Health and Development, London School of Hygiene and Tropical Medicine, London, WC1H 9SH UK; 3 Centre for Mathematical Modelling of Infectious Disease and Department of Infectious Disease Epidemiology, London School of Hygiene and Tropical Medicine, London, WC1H 9SH UK; 4 School of Life Sciences and Zeeman Institute for Systems Biology and Infectious Disease Epidemiology Research (SBIDER), University of Warwick, Coventry, CV4 7AL UK

**Keywords:** Data integration, Applied mathematics, Bayesian inference, Dynamical systems, Numerical simulations, Stochastic modelling, Viral infection

## Abstract

Respiratory syncytial virus (RSV) is responsible for a significant burden of severe acute lower respiratory tract illness in children under 5 years old; particularly infants. Prior to rolling out any vaccination program, identification of the source of infant infections could further guide vaccination strategies. We extended a dynamic model calibrated at the individual host level initially fit to social-temporal data on shedding patterns to include whole genome sequencing data available at a lower sampling intensity. The study population was 493 individuals (55 aged < 1 year) distributed across 47 households, observed through one RSV season in coastal Kenya. We found that 58/97 (60%) of RSV-A and 65/125 (52%) of RSV-B cases arose from infection probably occurring within the household. Nineteen (45%) infant infections appeared to be the result of infection by other household members, of which 13 (68%) were a result of transmission from a household co-occupant aged between 2 and 13 years. The applicability of genomic data in studies of transmission dynamics is highly context specific; influenced by the question, data collection protocols and pathogen under investigation. The results further highlight the importance of pre-school and school-aged children in RSV transmission, particularly the role they play in directly infecting the household infant. These age groups are a potential RSV vaccination target group.

## Introduction

In 2015 the estimated respiratory syncytial virus (RSV) acute lower respiratory illness (ALRI) burden in children less than 5 years old was 33.1 million cases resulting in 118,200 (94,600–149,400) deaths. Over 90% of the estimated RSV burden was in developing countries^1^. A recent study across sites in 7 low-income and low-middle-income countries looking into the aetiology of severe and very severe pneumonia found that RSV is the single pathogen with the largest attributable fraction^2^. Infants below 6 months of age experience the most severe disease^3^. There are currently over 50 candidate vaccines against RSV at different stages of development with the most advanced being a maternal vaccine^4–6^.

RSV disease occurs in a seasonal pattern with most populations experiencing annual cycles^7–11^. The virus can be classified into two antigenically and genetically distinct groups (RSV-A and RSV-B) and consecutive seasons are not only characterized by a change in the dominant group, but also changes to the genotype composition within groups^11,12^. Though several studies have predicted maternal vaccination would be effective^13–15^, by extending the duration of protection by passive immunity early in life, the vaccination of older children has also been theorized as an effective alternative or complementary strategy by producing a herd immunity effect^16–19^. Elder and, particularly, school-going children have been shown in previous work to be associated with increased risk of infant (sibling) infection^20–23^—though no direct infection link between the older siblings and the infant was confirmed—and have been identified as drivers of the initial epidemic phase^24^. Identifying the role of different age and social groups in RSV transmission networks may provide further evidence for optimal vaccine target groups.

Previously, using data from a cohort study that followed household members for 6 months, we have attempted to identify the source of infant infection in the household. In a descriptive analysis of the social-temporal data, school-going siblings were frequently (73%) identified as index cases in household outbreaks where an infant was infected^23^. In a phylogenetic analysis of whole genome sequence (WGS) data from a subset of the household data, the household source of infant infection was definitively identified for just 4 of the 23 infant cases in the subset data, while 9 others were identified as index cases in household outbreaks^25^. An attempt to use shared minor variants obtained from deep sequencing failed to add further resolution to the transmission chains^26^. In a modelling study using only the social-temporal data, it was found that about half of all cases occurred through within household transmission^27^. Independently, these studies were unable to clearly determine who infected the infants with RSV and how infection spread once introduced in the household.

In this paper, we extend a previous modelling study^27^ to integrate social-temporal and WGS data to identify generalizable characteristics of RSV transmission chains at the household level. In doing so, we identify if data integration, and hence increased pathogen resolution, increases the precision with which model parameters are estimated or changes the estimates such that different transmission dynamics are inferred. To our knowledge, this is the first attempt at combining these two data types in a single modelling framework for RSV. There are several approaches to integrating genetic data with other data types^28–30^, the choice of which is dependent on the data available and the aims of the study^31^. Similar to the approach used by Didelot et al.^32^, we use a two-step approach of first making inference from the genetic data and then incorporating this into the dynamic transmission model of RSV.

## Results

**Figure 1:**
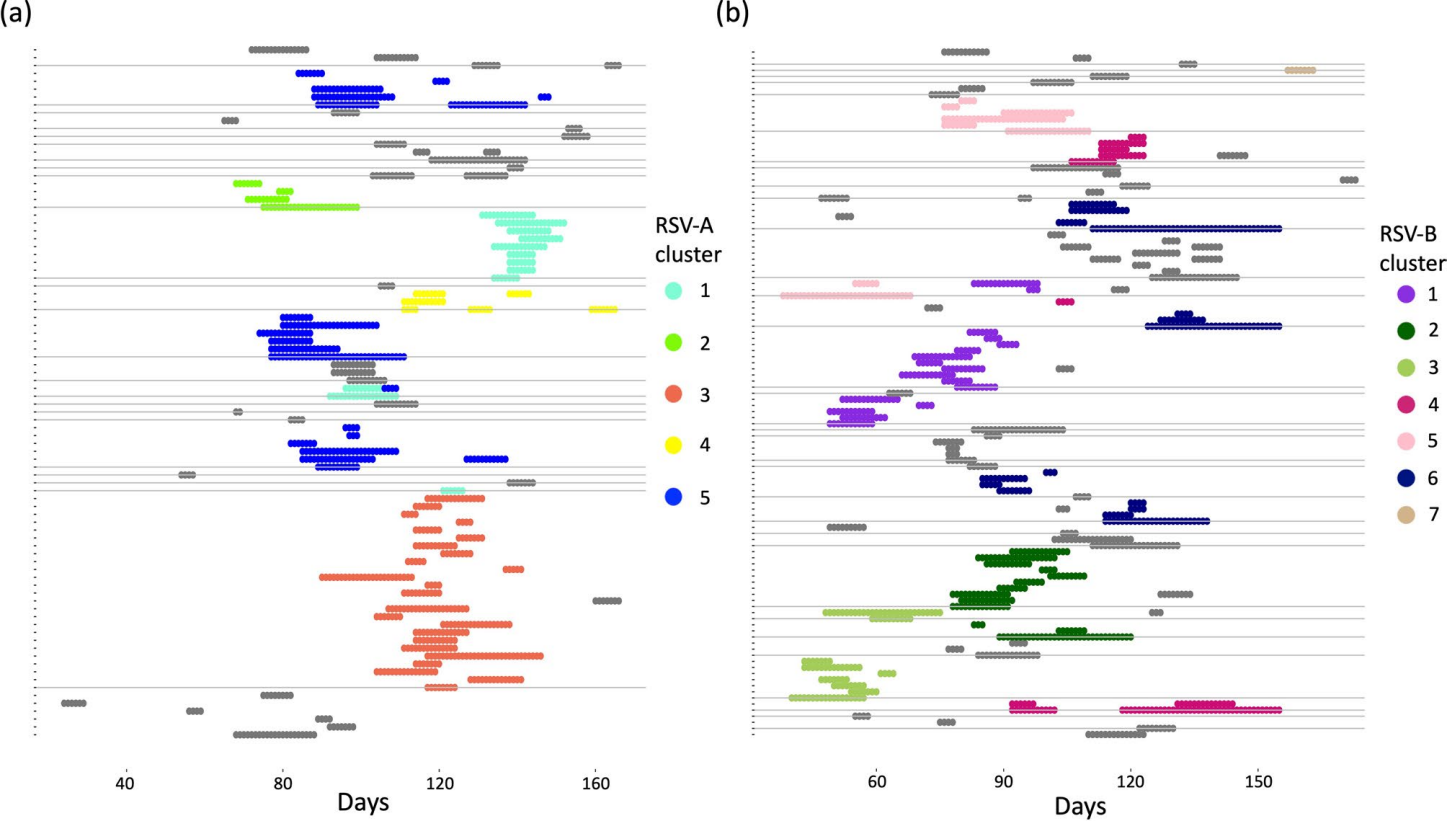
Shedding patterns for RSV-A, panel (**a**), and RSV-B, panel (**b**). Each notch on the y-axis represents a single individual and the horizontal grey lines separate the individuals into households, time in days is on the x-axis. The shaded regions show RSV shedding episode colour-coded by genetic cluster where grey represents episodes whose cluster id is unknown. This figure was generated using R programming language version 4.0.3^46^.

The data imputation process resulted in shedding episodes that ranged from 2 to 35 days for RSV A, and 3 to 45 days for RSV B. The cluster IDs for 12 of 43 RSV A episodes and 19 of 71 RSV B episodes with no genetic information were imputed prior to model fitting, the rest were inferred along with model parameters. The shedding patterns after the data pre-processing are shown in Fig. 1.

### Transmission dynamics inference

**Figure 2:**
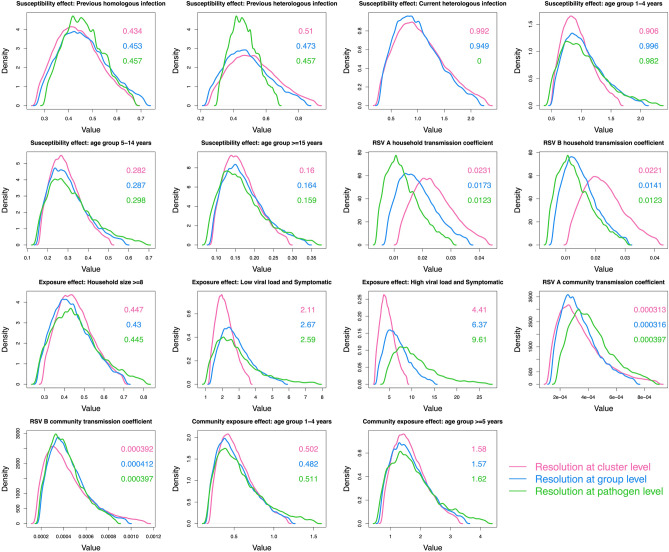
A comparison of the parameter distributions obtained from the model using different resolutions in pathogen identification. The green curves show the results using data at the pathogen level, the blue curves show the group level and the pink curves show the cluster level. Each panel shows 1 of 15 comparable parameters. The values in the panel are the median parameter estimates colour-coded by pathogen resolution. This figure was generated using R.

We compared the distributions of parameters estimated using RSV cases identified at the pathogen, group and cluster level in order to assess the impact of increased resolution in pathogen identification on estimated parameters. Fitting the model to group-level data is comparable to our previous approach^27^, details of the changes to the model equations without genetic clusters are provided in supplementary appendix A1. Figure 2 shows the comparison density plots of 15 comparable parameters across the model when fit to different pathogen resolutions.

For most of the parameters, except those measuring the effect of viral load and symptoms on infectiousness, increased pathogen resolution did not translate to increased precision in parameter estimates. Using cluster-level pathogen identification, symptomatic cases were inferred to be more infectious than asymptomatic cases, more so with a high viral load [relative infectiousness 4.4 (95% CrI:1 0.8, 9.0)] than a low viral load [relative infectiousness: 2.1 (95% CrI: 1.2, 3.7)]. Increased pathogen resolution did not alter the inference made on the effect of the different covariates on the rate of exposure. Based on the parameters estimated with cluster-level pathogen identification, we inferred an inverse relationship between age and susceptibility to exposure: relative to infants (< 1 year old), the percentage reduction in the rate of exposure for 1–4, 5–14 and ≥ 15 year olds was 9.4% (95% CrI: − 64%, 48.5%) , 71.8% (95% CrI: 49.1%, 83.7%) and 84% (95% CrI: 71.1%, 90.9%), respectively. RSV conferred partial immunity following infection, more so for homologous (56.6% (95% CrI: 32.7%, 73.3%) reduced exposure) than heterologous (49% (95% CrI: 9.4%, 74.3%) reduced exposure) group reinfections. Households of $\ge 8$ individuals had a 55.3% (95% CrI: 30.1%, 70.6%) reduction in pair-wise rate of exposure within the household relative to smaller households. The effects of age in community exposure and ongoing heterologous group infection on susceptibility were unclear as the parameters were estimated with 95% credible intervals including 1. Despite some households having similar genetic clusters, this similarity could not be explained by between household transmission. The estimated spatial distance kernel did not support any transmission events between members of different household present in these data. We assumed an exponential relationship between pair-wise nucleotide distance and the probability of a transmission event having occurred. The estimate for the decay rate was so small, $\vartheta$ = 0.000244 (95% CrI: 0.000000883, 0.00373), such that cases in the same genetic cluster could not be disentangled any further.

Increased pathogen resolution resulted in increases in the within household transmission coefficients for both RSV-A and RSV-B, and slight decreases in the values of the community transmission coefficient for RSV-B. These shifts in estimated distributions imply that with greater resolution on the infecting pathogen that each case is carrying, more infection events could be attributed to within household transmission than community transmission.

Parameter trace plots, results of convergence checks and the full parameter table from fitting to cluster-level data, can be found in supplementary appendix A2. To validate the model, we simulated multiple epidemics which verified that key aspects of the epidemic were being reproduced by the simulations. Details of this can be found in supplementary appendix A3.

### Highest probability transmission source (HPTS)

**Figure 3:**
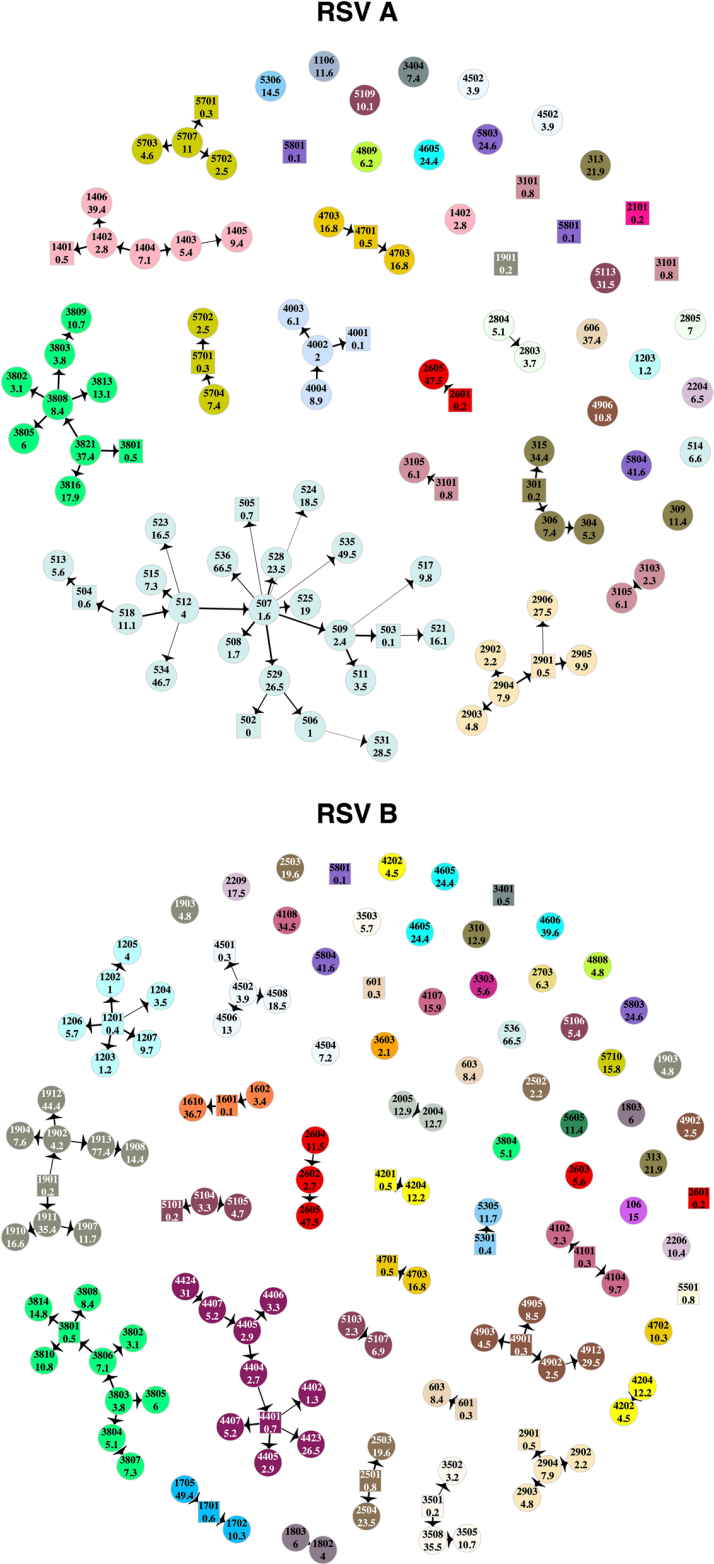
Transmission networks showing the highest probability source of transmission given by our model results. Each vertex is an RSV case labelled by individual study number (top) and age in years (bottom) and color-coded by household. Cases that are < 1-year-old are represented by square shaped vertices. The width of the connecting edge is proportional to the frequency at which the particular source was identified as the HPTS given different parameter set values. This figure was generated using R.

**Figure 4:**
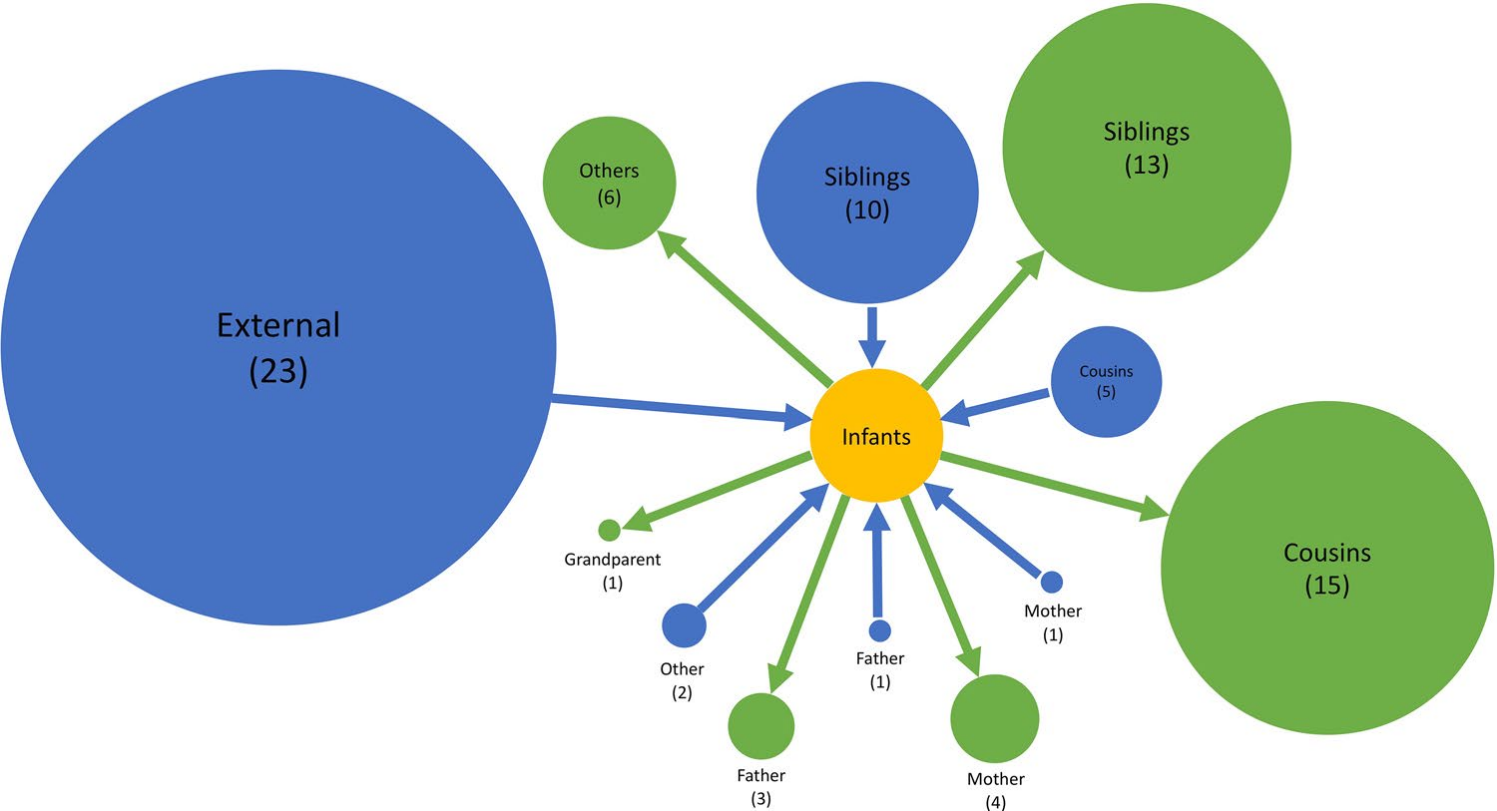
Network showing the sources of infection to the infant and who the infants infected as identified by social relationship. The blue circles show the sources to infant infection while the green show who the infants infected. The size of the circles is proportional to the number of cases which is given in brackets.

The HPTS was established for each case and these are shown in Fig. 3. Thirty-nine out of ninety-seven (40%) of the RSV-A and 60/125 (48%) RSV-B cases were from sources outside of the household; 33% (13/39) of RSV-A introductions into the household led to infection of other household members, as did 38% (23/60) of RSV-B introductions. Table 1 gives the age distribution of all index cases compared to the age distribution of index cases that led to other infections in the household (household outbreaks). A larger proportion of index cases that resulted in onward transmission were symptomatic (30/36), compared to those that did not (28/63). Household outbreaks were as frequently initiated by a symptomatic infant as they were by a symptomatic child between 5 and 13 years. Fifty five percent (11/20) RSV-A and 36% (8/22) RSV-B infant (< 1-year-old) infections were acquired within the household. Of the 11 infant RSV-A cases, 8 were infected by children aged between 2 and 13 years (5 siblings and 3 cousins), 1 was infected by another younger infant (cousin), 1 by a 16-year old (unknown relation) and 1 by a 37-year old (mother). Five out of 8 of the infant RSV-B cases were infected by children between 2 and 13 years (4 siblings and 1 cousin), 2 were infected by a 16-year-old (unknown relation) and 18-year-old (sibling) while one was most likely infected by a 49-year-old (father). Figure 4 shows the transmission network by relationship centred around the infants. Infants infected several household members, mostly siblings and cousins.

**Table 1:** Age distribution of index cases of household outbreaks. Index cases are clustered into 4 age groups and according to whether they led to onward transmission in the household or not**.**

Age group	RSV A		RSV B	
	No. index cases (number of symptomatic cases)	No. index cases leading to onward transmission (number of symptomatic cases)	No. index cases (number of symptomatic cases)	No. index cases leading to onward transmission (number of symptomatic cases)
< 1	9 (8)	3 (3)	14 (14)	9 (9)
1–4	5 (3)	1 (1)	12 (8)	6 (4)
5–13	16 (10)	7 (7)	18 (9)	5 (5)
≥ 13	9 (3)	2 (1)	16 (3)	3 (0)
Total	39 (24)	13 (12)	60 (34)	23 (18)

## Discussion

We carried out an analysis of longitudinal data on the social-temporal and genetic pattern of spread of RSV in a sample of households in rural Kenya with the aim of using data integration techniques to enhance our understanding of factors that influence infection onset and identifying characteristics of household transmission chains. We found that most household outbreaks were initiated by a symptomatic child < 13 years old, with infants and children aged 5–13 years contributing equally. Infant infections that occurred in the household were mostly attributed to transmission from an elder sibling or cousin between 2 and 13 years old. Similar to a simulation study based on the same population from which our data were collected^18^, we found over half of the infant infections were acquired outside of the household. Infants were the source of infection in 42/123 infections that were acquired in the household. Infants are therefore not only an important risk group but are also important as transmitters of household RSV infections. These results imply that a reduction in infant infections, say through a vaccine, would have a positive indirect (otherwise called herd) effect on RSV infections in other age groups. In addition, vaccination of household co-occupants of pre-school and school-going age would have an impact through reducing within household transmission to the infant. A significant portion of index cases that led to onward transmission in the household were symptomatic, a factor which we inferred to increase infectiousness. This implies—similar to past work^17^—that a vaccine that works against symptomatic infections, therefore reducing infectiousness, should be highly effective.

Through combining epidemiological and phylogenetic inference, our method was able to better resolve transmission chains within households compared to a preceding phylogenetic analysis^25,33^. The networks inferred from the present analysis did not contradict any of the inference from the phylogenetic analysis, with one exception. We assigned individual 3806 as the source of 3801’s RSV-B infection rather than 3805. In addition to considering the social grouping, temporal clustering of cases and genetic clusters, our approach also considers the infectiousness of a potential source. In this case, 3806 had symptoms and a high viral load in the three days preceding shedding onset in 3801, while 3805 did not. Such an example highlights the strength in our technique in being able to incorporate all possible determinants of a transmission event. It is worth mentioning that several super-spreader events were inferred. The model arrives at these networks based on the patterns in the available data. Though such events are plausible, to tease apart true super-spreader events from “convenience” networks, additional data on within household contacts would be needed, such as the kind collected by Kiti et al.^34^.

We found that increased pathogen resolution by including WGS data had a slight effect on both accuracy (resulting in narrower credible intervals for some parameters) and model inference (resulting in a change of transmission hypothesis). Relative to our previous work where we fit an individual based model to pathogen identification at the RSV-group level^27^, we narrowed down the relative infectiousness of symptomatic cases, from 2–7 to 2–4 times more infectious than asymptomatic cases. High viral load increased the infectiousness of symptomatic cases. We had previously hypothesized possible niche separations between RSV-A and RSV-B based on overlapping but slightly different distributions of the transmission coefficients. Increased pathogen resolution resulted in a slight change in estimated parameter distributions, as shown in Fig. 2, and this form of evidence was lost. Other inferred dynamics such as the effect of age, household size, previous infection remained relatively unchanged.

The lack of a drastic effect of increased pathogen resolution could be due to a lack of sufficient within-cluster genetic variation between cases and the study design. We introduced a genetic weight, with an exponential function form, to allow for cases within the same genetic cluster to be further disentangled. The maximum genetic distance between any pair of cases in the same cluster was 35 nucleotides in RSV-A, with most having less than 10 nucleotides difference. The variation was even smaller for RSV-B sequences, with a maximum within-cluster genetic distance of 17 nucleotides. This led to the genetic weight being assigned to every pair of cases being close to 1, making every transmission event equally likely regardless of within-cluster genetic distance. With regard to study design, the frequency in sampling, increased the accuracy of inferred onset dates, while, information on the social structuring of the population in the form of households provided information on some of the most frequent contacts each participant had. Since the genetic information’s clustering pattern mimicked the household structure, its utility was likely marginal. This result, though unanticipated, should not be surprising; Campbell et al.^35^, in integrating genetic, temporal and contact data found that contact data could replace the genetic data in transmission chain inference. This implies that good quality data on timing of cases and their most frequent contacts is key to be able to infer transmission characteristics. Nonetheless, it should be borne in mind that during an outbreak, it can be difficult to effectively gather contact data. In place of a dense sampling, integrating temporal and genetic data is the next best thing. Our results point to data integration being able to reduce measurement error (increase accuracy of parameter estimates) and provide information essential for correct inference (change interpretation of estimated parameters).

We inferred that 55% of infant RSV-A infections were acquired within the household, compared to 36% of infant RSV-B. There were also slight differences between the RSV groups in terms of proportion of cases that were index cases and proportion of index cases that led to onward transmission. These differences between RSV-A and RSV-B might be specific to the outbreak under investigation, however, there is pre-existing evidence of differences in mutation rates^36,37^ and transmissibility^38^ which could explain our observations.

This study is not without its limitations. Firstly, similar to previous work^32,39,40^, we used a two-step approach in our application of phylodynamics. This has the potential to lead to inconsistencies that would otherwise not occur with simultaneous inference of the evolutionary and epidemiological dynamics. However, given that we only used aggregated results of the phylogenetic analysis, in the form of clusters, and raw nucleotide distances as opposed to phylogenetic tree distances, we do not heavily rely on the exact results of the independent phylogenetic analysis. Using genetic clusters provides the advantage of being able to identify obvious separate introductions, a characteristic that can be difficult to account for in the models of simultaneous inference. The two-step approach was more computationally tractable than a simultaneous-inference version of it would have been. Secondly, the clusters were not probabilistically determined, in particular, uncertainty in the estimated date of sequence divergence was not considered. Finally, given the sampling interval of 3–4 days, short duration shedding episodes might have been missed and apparent co-index cases might actually have different onset dates.

In conclusion, we were able to integrate the results of a phylogenetic analysis with epidemiological data to infer that nearly half of the RSV infections in this study were acquired within the household. We showed explicitly that most infants were infected by an older sibling or cousin (2–13 years). A vaccine that limits the transmission capabilities (e.g. by eliminating ARI symptoms and reducing viral load) of this age group is therefore likely to reduce a significant portion of infant infection through indirect protection. The differences in infection patterns and interaction through modified susceptibility inferred between RSV-A and RSV-B warrant further investigation.

## Methods

### Data

During a seasonal RSV outbreak beginning late 2009, members of 47 households in a rural location of coastal Kenya were followed up for a period of 6 months with an aim of recording the incidence of RSV and inferring who infects the infant^23^. A household in this study was defined as comprising of people who share food from the same kitchen. Households were recruited on the basis of having an infant born after the previous RSV epidemic who had at least 1 elder sibling < 13 years old. Households in the study had a median size of 11 members (inter-quartile range: 8, 19). Members of the household had nasopharyngeal swab (NPS) samples and clinical data collected every 3–4 days. The samples were tested for RSV using an in-house real-time multiplexed polymerase chain reaction (rtPCR) assay^41^. A sample was considered RSV positive if the rtPCR cycle threshold value was ≤ 35. An RSV infection episode was defined as a period within which an individual provided positive samples for the same RSV group that were no more than 14 days apart. A shedding episode was referred to as symptomatic if, within the window of virus shedding, there is at least one day where symptoms were recorded. The symptoms of interest are those of an acute respiratory illness (ARI), which are: cough, or nasal discharge/blockage, or difficulty breathing. There were 16,928 samples collected, of which 205 were positive for RSV-A and 306 for RSV-B. This translated to 97 RSV-A episodes (88 infected individuals and 25 infected households) and 125 RSV-B episodes (113 infected individuals and 34 infected households).

Positive samples from 20 households were targeted from genome sequencing on the basis of households having $\ge 2$ members infected. From 415 positive samples, 374 were processed for sequencing using the the QIAamp viral RNA extraction Kit (QIAGEN, Hilden, Germany) for RNA extraction and the Illumina MiSeq platform to generate short reads. Reads passing quality checks were de novo assembled into longer contigs using SPAdes v3.5.0^42^. Two hundred and forty-six samples were successfully amplified and assembled, 191 of which had contigs > 14,000 nucleotides (> 90% of the RSV genome). All 246 genomes are accessible on GenBank under the accession numbers MH594350—MH594461 for the RSV-B genomes and KX510136-KX510266 for the RSV-A^25^. For the present analysis, we utilise the 191 near-complete genomes—referred to as whole genome sequences (WGS)—obtained from 98 infected individuals. The median number of sequences per individual was 2 (IQR: 1–2)). The sequences were distributed across 103 (41.2%) samples, 54 (55.6%) episodes, 50 (56.8%) individuals and 9 (36%) households for RSV-A; 88 (28.8%) samples, and 54 (43.2%) episodes, 53 (45.9%) individuals and 15 (44.1%) households for RSV-B. During phylogenetic analysis, as described previously^25^, genetic clades and subclades were established based on a combination of criteria: nucleotide distance cut-off, clustering patterns on the global RSV phylogeny and the inferred date of sequence divergence. Viruses were grouped in the same clade if they occurred as a monophyletic group on the global phylogeny, had < 60 pairwise SNPs across the genome with every other member of that clade and diverged more than a year prior to their date of collection. Within clades, viruses formed a sub-clade if they showed > 10 pairwise SNPs differences across the genome and were estimated to have diverged more than six months prior to their date of collection. We did not make a distinction between clades and subclades, resulting in 5 RSV-A and 7 RSV-B clusters.

Informed written consent was obtained from all the study participants or their parent/guardian. The KEMRI-Scientific and Ethical Review Committee in Kenya provided ethical approval for the initial study and any analysis thereafter. The Observational/ Interventions Research Ethics Committee at the London School of Hygiene and Tropical Medicine provided further approval for this analysis. All study procedures were performed in accordance with the approved protocol guidelines and in compliance with the relevant regulations.

### Transmission model

#### Model description

We took a sequential approach to making inference from the genetic and epidemiological data described. The primary aim of the model was to infer the determinants of infection in individual hosts. Exposure events are not observed, however, temporal, social (household) and genetic clustering patterns of shedding episodes could aid to narrow down potential exposure windows. Similar to our previous work^27^, we used an individual based model of RSV-A and RSV-B transmission and calibrated it to social-temporal and genetic data.

Everyone is assumed to be uninfected and susceptible to infection by RSV at the start of the outbreak, but the risk of infection was dependent on age. Once individuals were exposed to infection, they entered a latency period that ranged between 2 and 5 days after which they became infectious^43^. After the infectious period, individuals became susceptible to infection again, but with a modified risk, i.e. RSV conferred partial transient immunity that lasts as long as the outbreak is ongoing. This partial immunity is assumed to be different for heterologous and homologous RSV group re-infections. Individuals can get heterologous group co-infections, hence, we explored if susceptibility to infection by RSV-A was modified if an individual was currently shedding RSV-B, and vice-versa.

The main assumptions about transmission are contained in the equation giving the per capita rate at which individuals are exposed to infection, $\lambda$. In order to incorporate the genetic information, we expressed $\lambda$ as a daily (index ***t***), genetic-cluster-specific (index ***c***) per capita (index ***i***) rate, denoted $\lambda_{i,c} \left( t \right)$. At its base:

$$\lambda_{i,c} \left( t \right) = contact\;rate*probability\;of\;transmission\;given\;contact*number\;of\;infectious\;contacts\left( t \right)$$

$$\begin{aligned} \lambda_{i,c} \left( t \right) & = baseline\;rate\;of\;exposure*number\;of\;infectious\;contacts\left(t\right) \\ & = \eta *\mathop \sum \limits_{{\begin{array}{*{20}c} {j \in infectious} \\ {contact} \\ \end{array} }} I_{j,c} \left( t \right) \\ \end{aligned}$$

where $\eta$ is the baseline rate of exposure and $I_{j,c} \left( t \right)$ is an indicator variable of infectiousness of contact ***j*** at time ***t***.

The individuals in our model are grouped into households and we allow for exposure to infection to occur within or outside the household. Therefore, the basic rate of exposure is decomposed into 2 parts, a within-household and community rate of exposure.

$$\begin{aligned} \lambda_{i,c} \left( t \right) & = \left( {\eta *\mathop \sum \limits_{{\begin{array}{*{20}c} {j \in infectious} \\ {household} \\ {contact} \\ \end{array} }} I_{j,c} \left( t \right)} \right) + \left( {\varepsilon *\mathop \sum \limits_{{\begin{array}{*{20}c} {j \in infectious} \\ {community} \\ {contact} \\ \end{array} }} I_{j,c} \left( t \right)} \right) \\ \eta & = baseline\; rate\;of\;within\;household\;exposure \\ \varepsilon & = baseline\;rate\;of\;community\;exposure \\ \end{aligned}$$

In addition to the assumptions about RSV natural history, we extend this basic formulation to explore if factors such as household size, infectiousness (as determined by the viral load proxy measurement of rtPCR cycle threshold (Ct) and symptoms of acute respiratory illness (ARI)) and age are determinants of exposure. The form for $\lambda_{i,c} \left( t \right)$, that now includes a household index ***h***, is given by:

1 $$\lambda_{i,h,c} \left( t \right) = S_{i,g} \left( t \right)\left[ {M_{i,h} \left( t \right)\mathop \sum \limits_{j \ne i} HH\_Rate_{h,c,j \to i} \left( t \right) + Comm\_Rate_{i,c} \left( t \right)} \right]$$

where

$M_{i,h} \left( t \right)$ is a binary indicator variable for recorded presence in the household.

$S_{i,g} \left( t \right)$ is the factor modifying exposure by recent group-specific infection history, age and group-specific shedding status at time ***t*** given by:

1.1 $$S_{i,g} \left( t \right) = \exp \left( {\phi_{Y,hist} \left( {Infection\_History_{i} \left( t \right)} \right) + \phi_{X,age} \left( {Age\_group_{S,i} } \right) + \phi_{W,curr} \left( {Shedding\_status_{i} \left( t \right)} \right)} \right)$$

$HH\_Rate_{h,c,j \to i} \left( t \right)$ is the cluster specific within household exposure rate from infectious individual *j* present in the household at time *t*, and is given by:

1.2 $$HH\_Rate_{h,c,j \to i} \left( t \right) = \eta_{g} \times \psi_{H} \left( {Household\_size_{i} } \right) \times \psi_{I,inf} \left( {Infectivity_{j,h,c} \left( t \right)} \right) \times M_{j,h} \left( t \right)$$

$Comm\_Rate_{i,c} \left( t \right)$ is the cluster specific community (external to the household) exposure rate given by:

1.3 $$Comm\_Rate_{i,c} \left( t \right) = \varepsilon_{g} \times \psi_{E,age} \left( {Age\_group_{E,i} } \right)\left( {\left( {M_{i,h} \left( t \right)\mathop \sum \limits_{{\begin{array}{*{20}c} {j \ne i, j not in} \\ { i^{\prime}s house} \\ \end{array} }} Sampled\_Neighbour\_Rate_{h,c,j \to i} \left( t \right)} \right) + f_{c} \left( t \right)} \right)$$

The community rate is further decomposed into two components to account for possible distant-dependent transmission between households in the dataset ($Sampled\_Neighbour\_Rate_{h,c,j \to i} \left( t \right)$), and from unknown sources represented by a background function ($f_{c} \left( t \right)$). Further details of the exact formulations can be found in supplementary appendix A4. Table 2 gives a brief description of the parameters in the presented equations, all of which were estimated.

**Table 2:** Model parameters and their descriptions.

Parameter (symbol)	Description
$\phi_{Y}$	Coefficients modifying susceptibility to infection by a particular RSV group depending on infection history
$\phi_{X}$	Coefficients modifying susceptibility to RSV depending on age
$\phi_{W}$	Coefficient modifying susceptibility to a particular RSV group based on shedding status of the heterologous group type
$\eta_{g}$	Baseline rate of within household exposure by RSV group, per person per day
$\psi_{H}$	Coefficient modifying within household exposure by household size
$\psi_{I}$	Coefficients modifying infectiousness by viral load and symptom status
$\varepsilon_{g}$	Baseline rate of community exposure by RSV group, per person per day
$\psi_{E}$	Coefficients modifying community exposure by age group

#### Linking the model to data

To link the rate of exposure equation to the observed data, we nested this equation within the probability of infection onset given exposure, $p_{i,h,c}$. We first defined the probability of exposure:

2 $$\alpha_{i,h,c} \left( t \right) = \left( {1 - exp^{{ - \mathop \sum \limits_{{C^{\prime}}} \lambda_{i,h,c} \left( t \right)}} } \right)*\left( {\frac{{\lambda_{i,h,c} \left( t \right)}}{{\mathop \sum \nolimits_{{C^{\prime}}} \lambda_{i,h,c} \left( t \right)}}} \right)$$

where $C^{\prime}$ is the set of all clusters in a given RSV group.

Assuming that the duration of latency can range from 0 to 5 days with probabilities [0, 0,0.33,0.33,0.25,0.083]^43^, we then have the following probability of onset at time *t* given no onsets or shedding until *t*:

3 $$p_{i,h,c} \left( t \right) = \mathop \sum \limits_{l = 0}^{L} \theta_{l} \alpha_{i,h,c} \left( {t - l} \right)$$

where L is the maximum latency period and $\theta_{l}$ is the probability that the latency period is exactly $l$ days.

For each individual we then assumed that their onset event (or lack thereof) on a given day ***t*** was determined by a Bernoulli distribution with probability $p_{i,h,c} \left( t \right)$. To incorporate the information on genetic clusters, we introduced a genetic weight, $P_{j \to i}$, on the rate of exposure from a given case in the same household, $HH\_Risk_{h,c,j \to i} \left( t \right) \times P_{j \to i}$, or from a sampled neighbour, $Sampled\_Neighbour\_Risk_{h,c,j \to i} \left( t \right) \times P_{j \to i}$. For

$$d_{gen} \left( {i,j} \right) = pairwise\;nucleotide\;distance\;between\;case\;i\;and\;case\;j$$

$$P_{j \to i} = exp^{{ - d_{gen} \left( {i,j} \right)*\vartheta }}$$

where $\vartheta$ is estimated along with other model parameters. With this formulation, the larger the nucleotide distance between a suspected transmission pair, the less likely that an infection event occurred. Further details of this can be found in supplementary appendix A4.

**Figure 5:**
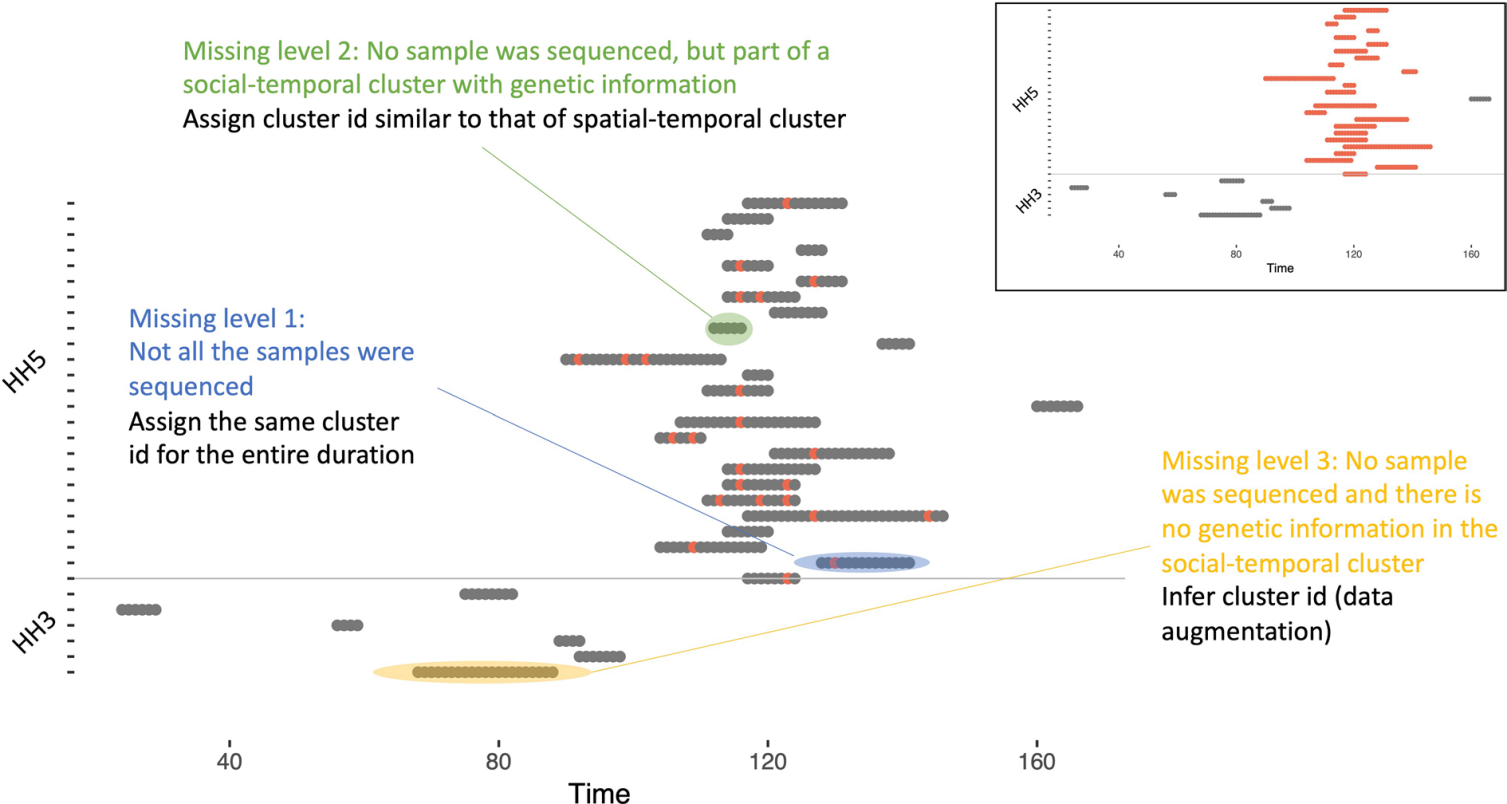
An illustration of the genetic cluster imputation process. Each notch on the y-axis represents a single individual in a household and the horizontal grey line separates the data from two households. Time in days is shown on the x-axis. Each shaded horizontal region shows an imputed shedding episode with the coloured dots showing the days in the episode where there is genetic cluster information. Inset: the results of imputing genetic information at missing level 1 and 2. This figure was partly generated using R.

The model requires daily infection data where a viral shedding episode can be identified by RSV group and by genetic cluster within each group. However, given the sampling interval and sequencing of < 50% of the samples, we had to make assumptions to fill in the days of missing data. We first imputed complete shedding durations by assuming that shedding started half-way in-between the last negative and the first positive samples and ended half-way in-between the last positive and the first negative samples. Subsequently, we imputed the genetic cluster ID for episodes that had at least one sequence (missing level 1), and for episodes with no sequences yet were part of a household outbreak with at least one sequence (missing level 2). A household outbreak is defined as overlapping shedding episodes in the same household such that there is at least one individual with detectable virus on any day during the course of the outbreak. The cluster ID for any episodes with no sequences that were not part of household outbreak with at least one sequence (missing level 3) were inferred along with the model parameters. The imputation of genetic clusters is illustrated in Fig. 5 and further details of the data pre-processing can be found in supplementary appendix A5.

We use Bayesian inference and Metropolis-Hasting Markov Chain Monte Carlo (MH-MCMC) to obtain estimates of the model parameters and augment missing cluster identities given the observed data. Further details can be found in supplementary appendix A6. All the computation was done using the *Julia* language (version 1.1)^44,45^. The code is publicly available at https://github.com/KadzoK/HH-Transmission-Model-2015-2020-/tree/master/Part_2.

#### Transmission chain inference

The estimated parameter distributions were then used to determine infection sources for every case. A single parameter set was obtained by randomly selecting a position in the chain of posterior samples. Given a parameter set, potential infection sources are identified for each ***case i*** based on who was shedding within 5 days of shedding onset in ***case i***. The probability of the observed onset given a particular source is calculated for all the potential sources, the one with the highest value—the highest probability transmission source (HPTS)—is selected as the source associated with the particular parameter set. We sampled 100 parameter sets and established the HPTS each time. From the distribution of 100 HPTS, the one with the highest frequency was selected as the source of transmission. This frequency becomes the weight assigned in the transmission network. Further details can be found in supplementary appendix A7.

## Supplementary Information

Supplementary Information.

## Data Availability

All the genomes used in the phylogenetic analysis are accessible on GenBank under the accession numbers MH594350–MH594461 for the RSV-B genomes and KX510136-KX510266 for the RSV-A^25^. The household data used to derive the social-temporal shedding patterns and more detailed information beyond the metadata provided can be requested through Havard Dataverse (https://doi.org/10.7910/DVN/BPSZZS/1MO6TO).
