# Supplementary material for: Integrating epidemiological and genetic data with different sampling intensities into a dynamic model of respiratory syncytial virus transmission
Source: Sci Rep. 2021 Jan 14;11:1463. doi: 10.1038/s41598-021-81078-x (PMC7809427; doi:10.1038/s41598-021-81078-x)
Supplement: Supplementary file 1 — Supplementary Information. [file 41598_2021_81078_MOESM1_ESM.pdf]

Supplementary appendix

Integrating epidemiological and genetic data with different sampling intensities into a dynamic model of respiratory syncytial virus transmission

Ivy K. Kombe[1*], Charles N. Agoti[1], Patrick K. Munywoki[1], Marc Baguelin[3], D. James Nokes[1,4], Graham F. Medley[2]

[1] KEMRI-Wellcome Trust Research Programme, KEMRI Centre for Geographical Medical Research-Coast. P.O. Box 230-80108, Kilifi, Kenya.

[2] Centre for Mathematical Modelling of Infectious Disease and Department of Global Health and Development, London School of Hygiene and Tropical Medicine. London, WC1H 9SH, United Kingdom.

[3] Centre for Mathematical Modelling of Infectious Disease and Department of Infectious Disease Epidemiology, London School of Hygiene and Tropical Medicine. London, WC1H 9SH, United Kingdom.

[4] School of Life Sciences and Zeeman Institute for Systems Biology & Infectious Disease Epidemiology Research (SBIDER), University of Warwick. Coventry, CV4 7AL, United Kingdom.

*Correspondence: Ivy K. Kombe (ivkadzo@gmail.com)

Table of contents

A1. Model modification to fit pathogen data identified at group resolution 3

A2. Extra results...6

Parameter trace plots and convergence checks 6

A3. Model validation 16

A4. Further details of the transmission model 25

The community rate of exposure 25

The background community function 25

Linking the model to data (the likelihood function) 30

A5. Data pre-processing...32

Imputing complete shedding, ARI and presence durations. 32

Imputing missing genetic information 35

A6. Further details of the adaptive MH-MCMC algorithm 38

Choice of proposal distributions for the parameters 39

Pseudo algorithm for our implementation of MH-MCMC 40

A7. Further details of the HPTS 43

Establishing the highest probability transmission source (HPTS)...........................43

A8. References..46

A1. Model modification to fit pathogen data identified at group resolution

The model for fitting to group-level data is similar in structure to the model of sequence data presented in the main text, however, there is no identification of the infecting pathogen at the cluster level, only at the group level. The rate of exposure to a particular RSV cluster ***g*** acting on a susceptible person ***i*** from household ***h*** at time ***t***:

$$\lambda_{i,h,g}(t) = S_{i,g}(t)\left[M_{i,h}(t)\sum_{j\neq i} HH_{Rate_{h,g,j\to i}}(t) + \, Comm\_Rate_{i,g}(t)\right] \qquad \dots (Eq\ A1.1)$$

Where:

$S_{i,g}(t)$ is the factor modifying exposure by recent group specific infection history, age and group specific shedding status at time ***t*** given by:

$$S_{i,g}(t) = \exp\Big(\phi_{Y,hist}\big(Infection\_History_i(t)\big) + \, \phi_{X,age}\big(Age\_group_{S,i}\big) + \phi_{W,curr}\big(Shedding\_status_i(t)\big)\Big)$$

$HH\_Rate_{h,g,j\to i}(t)$ is the group specific within household exposure rate given by:

$$HH\_Rate_{h,g,j\to i}(t) = \eta_g \times \psi_H(Household\_size_i) \times \psi_{I,inf}\big(Infectivity_{j,h,g}(t)\big) \times M_{j,h}(t)$$

$Comm\_Rate_{i,g}(t)$ is the cluster specific community (external to the household) exposure rate given by:

$$Comm\_Rate_{i,g}(t) = \varepsilon_g \times \psi_{E,age}(Age\_group_{E,i}) \left(\left(M_{i,h}(t) \sum_{\substack{j\neq i,\ j\ not\ in \\ i's\ house}} Sampled\_Neighbour\_Rate_{h,g,j\to i}(t) \right) + f_g(t) \right)$$

Where:

$$Sampled\_Neighbour\_Rate_{h,g,j\to i}(t) = \psi_{I,g,j}(t) \times K(d_{i,j}, \kappa) \times M_{j,h}(t)$$

The background function $f_g(t)$ is derived the same way $f_c(t)$ is, as described in the main text.

Since we do not use genetic distances in this version of the model, we do not estimate ϑ for $P_{j\to i} = exp^{-d_{gen}(i,j)*\vartheta}$ or $P_{j\to i} = 1\ if\ d_{gen}(i,j) \leq \vartheta, 0\ otherwise$, making the total number of parameters 17.

Following from the rate of exposure is the probability of exposure give by:

$$\alpha_{i,h,g}(t) = \left(1 - exp^{-\lambda_{i,h,g}(t)}\right) \qquad \ldots (Eq\ A1.2)$$

The probability of onset is given as:

$$p_{i,h,g}(t) = \sum_{l=0}^{L} \theta_l \alpha_{i,h,g}(t-l) \qquad \ldots (Eq\ A1.3)$$

Where L is the maximum latency period and θ_l is the probability that the latency period is exactly l days.

The likelihood for individual ***i***'s data is given as:

$$L_i = \prod_g \left[\prod_{u \in U_{i,h,g}} p_{i,h,g}(u) \prod_{a \in A_{i,h,g}} \left(1 - p_{i,h,g}(a)\right) \right]$$

The total likelihood is thus given by the product of L_i over all the individuals in the data

$$L = \prod_i \left[\prod_g \left[\prod_{u \in U_{i,h,g}} p_{i,h,g}(u) \prod_{a \in A_{i,h,g}} \left(1 - p_{i,h,g}(a)\right) \right] \right]$$

A2. Extra results

Parameter trace plots and convergence checks

Three MCMC chains were run, and the burn-in point assessed for each, after which, the reminder of the three chains were combined to give the posterior estimates for the parameters presented as median and 95% credible intervals. The figures below show the evolution of the parameter value with increasing number of iterations for the model with pathogen identification at the genetic cluster level (cluster model) and at the group level (group model).

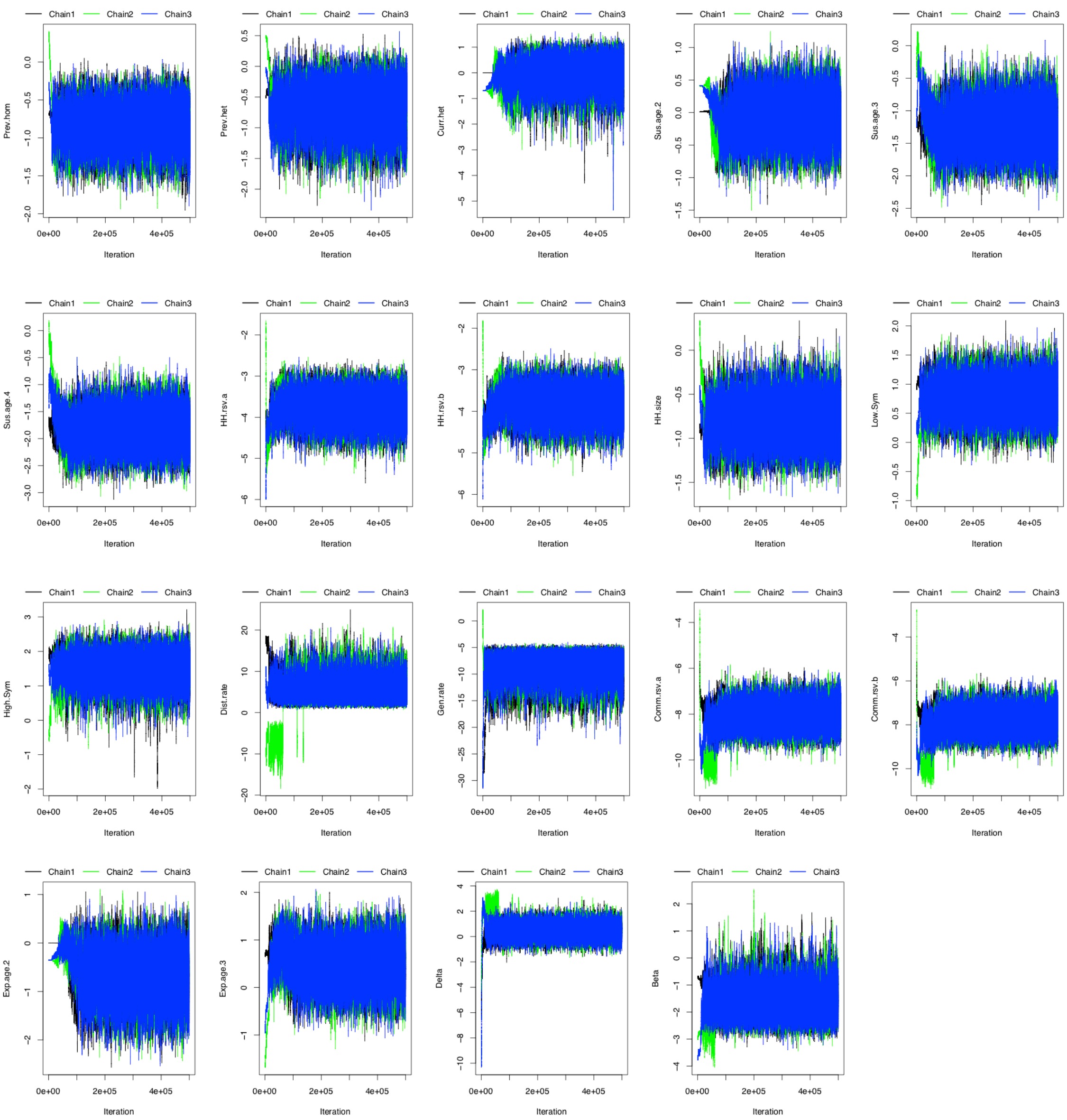

Figure A2. 1: Trace plots of parameters in the cluster model.

Three chains were initiated at different parameter values and these are shown in black (Chain 1), green (Chain 2) and blue (Chain 3) lines. The x-axis shows the iteration number, while the y-axis shows the log parameter value.

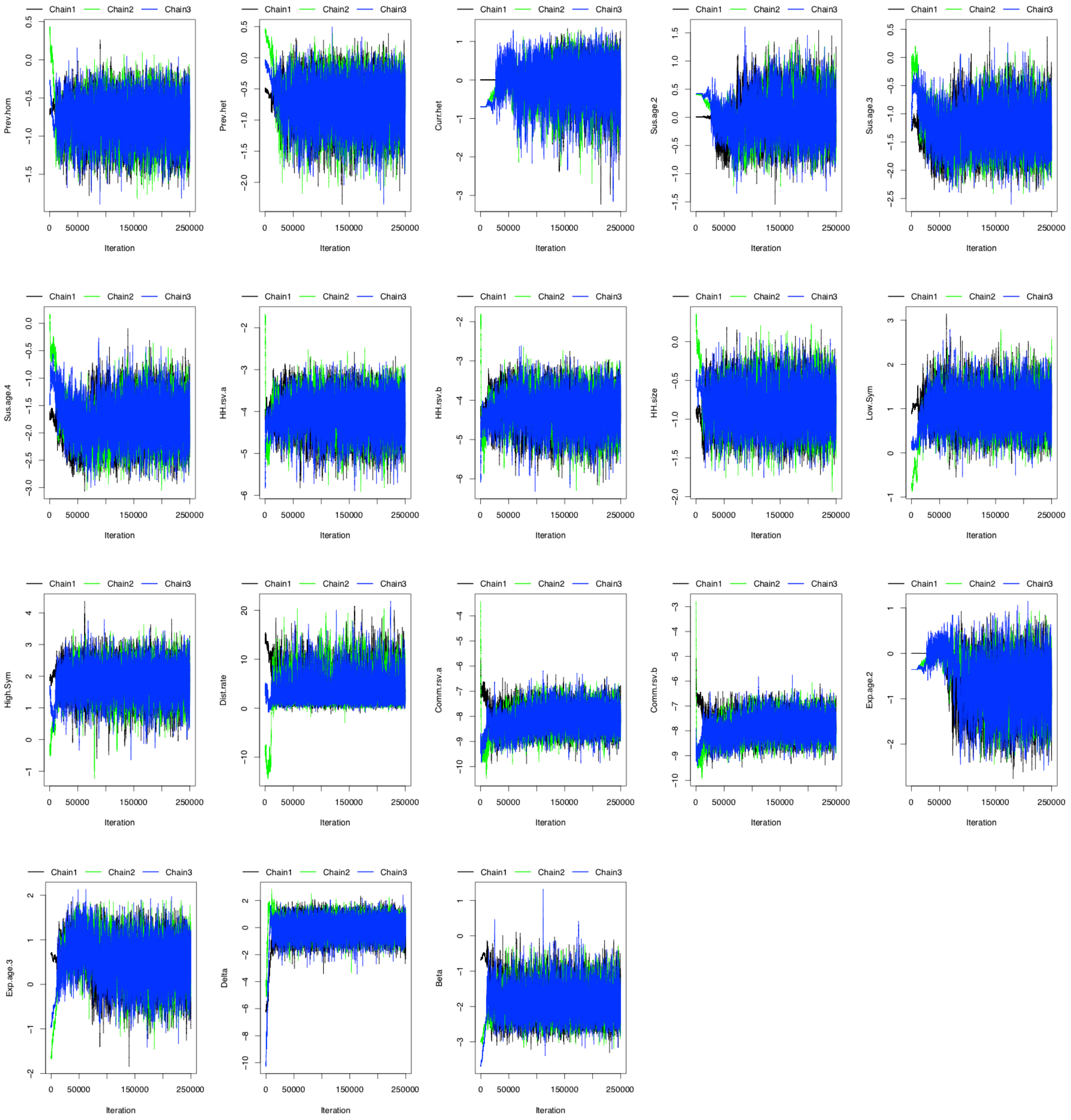

Figure A2. 2: Trace plots of parameters in the group level data model.

Three chains were initiated at different parameter values and these are shown in black (Chain 1), green (Chain 2) and blue (Chain 3) lines. The x-axis shows the iteration number, while the y-axis shows the log parameter value.

To confirm convergence observed in the trace plots, we calculated the Gelman-Rubin-Brooks statistic and the effective sample size. When using the GRB statistic, convergence is said to have occurred if the ratio of pooled/within chain variance is close to 1. The GRB

statistic assumes that the target distribution is Normal. The plot below shows the value of the GRB statistic as the number of iterations increases for each parameter. This is to check whether a value close to one was reached by chance or if the trend line had truly stabilized close to 1.

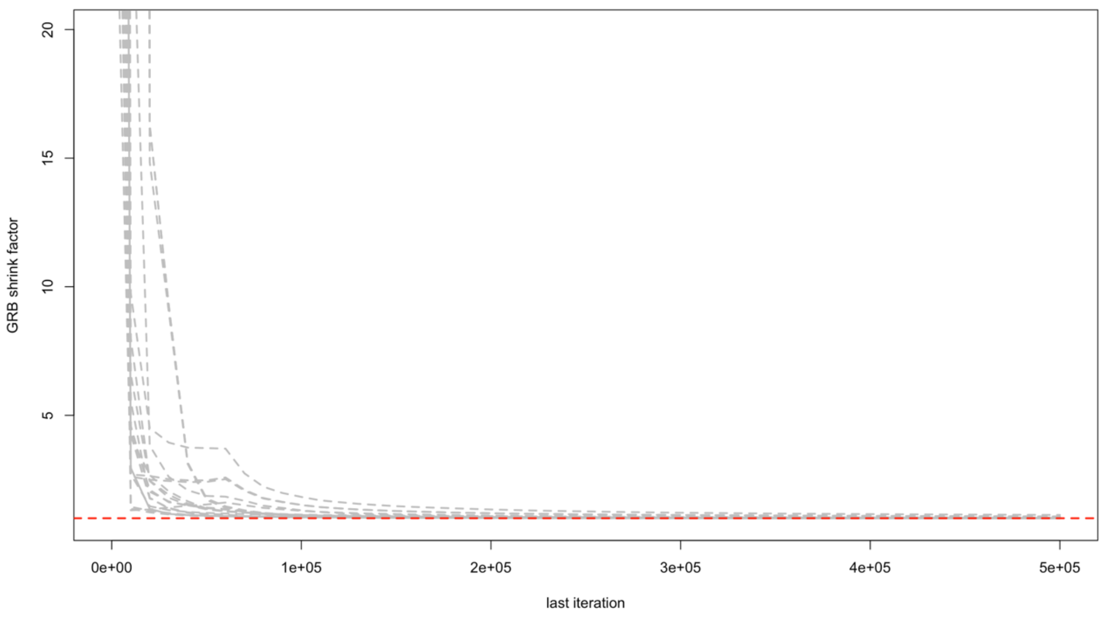

Figure A2. 3: The evolution of the Gelman-Rubin-Brooks (GRB) statistic (shrink factor) as the number of iterations increases.

Each grey line represents a model parameter in the cluster level data model and the dashed red line shows the value 1.

The point estimated of the GRB and the values of the ESS after burn in are given in the table below.

Table A2. 1: The value of the GRB statistic (to 3 significant figures) and the ESS after burn-in are shown for the parameters in the cluster level data model.

Parameter	Point estimate GRB statistic	ESS
Prev.hom	1	10607
Prev.het	1	10073
Curr.het	1.01	7131
Sus.age.2	1.01	9154
Sus.age.3	1.02	9771
Sus.age.4	1.02	10384
HH.rsv.a	1	9476
HH.rsv.b	1.01	9765
HH.size	1	10147
Low.Sym	1.02	9987
High.Sym	1.01	9774
Dist.rate	1.16	10455
Gen.rate	1.04	10436
Comm.rsv.a	1.09	7847
Comm.rsv.b	1.09	7823
Exp.age.2	1	8432
Exp.age.3	1.01	9863
Delta	1.04	7908
Beta	1.03	6678

The mGRB is 1.07 and the mESS is 10008.

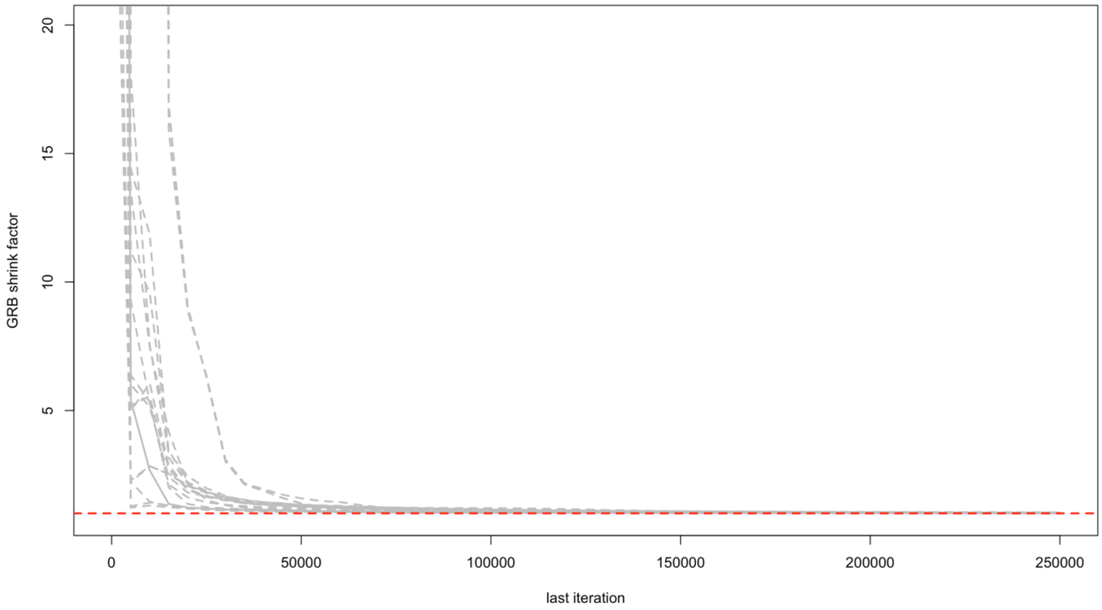

Figure A2. 4: The evolution of the Gelman-Rubin-Brooks (GRB) statistic (shrink factor) as the number of iterations increases.

Each grey line represents a model parameter in the group level data model and the dashed red line shows the value 1.

Table A2. 2: The value of the GRB statistic (to 3 significant figures) and the ESS after burn-in are shown for the parameters in the group level data model.

Parameter	Point estimate GRB statistic	ESS
Prev.hom	1.01	3713
Prev.het	1.02	3978
Curr.het	1.07	2309

**Sus.age.2**	1.02	2998
**Sus.age.3**	1.03	3617
**Sus.age.4**	1.04	3694
**HH.rsv.a**	1.01	3426
**HH.rsv.b**	1.01	3361
**HH.size**	1.02	3673
**Low.Sym**	1.04	3957
**High.Sym**	1.03	3744
**Dist.rate**	1.07	3374
**Comm.rsv.a**	1.05	4069
**Comm.rsv.b**	1.05	4093
**Exp.age.2**	1.02	2858
**Exp.age.3**	1.02	3476
**Delta**	1.04	5331
**Beta**	1.04	3873

The mGRB is 1.09 and the mESS is 4146.

As a rule of thumb, a GRB of <1.1 is generally considered good, as such, it is safe to conclude that there was convergence.

Table A2. 3: Median and 95% credible intervals for parameters estimated using the model with sequence data.

Symbol	Description	Name	Median (95% Credible interval)
ϕ_Y	Coefficients modifying susceptibility to infection by a particular RSV group depending on infection history. *Prev.hom* estimates the effect of a previous homologous group infection, and *Prev.het* the effect of a previous heterologous infection	*Prev.hom*<br>*Prev.het*	0.434 (0.267, 0.673)<br>0.510 (0.257, 0.906)
ϕ_W	Coefficient modifying susceptibility to a particular RSV group based on shedding status of the heterologous group type	*Curr.het*	0.992 (0.269, 2.30)
ϕ_X	Coefficients modifying susceptibility to RSV by age. *Sus.age.2* estimates modification to group 1-4 years, *Sus.age.3* 5-15 years and *Sus.age.4* ≥15 years relative to group <1 year.	*Sus.age.2*<br>*Sus.age.3*<br>*Sus.age.4*	0.906 (0.515, 1.64)<br>0.282 (0.163, 0.509)<br>0.160 (0.0909, 0.289)

η_g	Baseline rate of within household exposure by RSV group, per person per day.	*HH.rsv.a*<br>*HH.rsv.b*	0.0231 (0.0116, 0.0429)<br>0.0221 (0.0109, 0.0413)
ψ_H	Coefficient modifying the amount of within household exposure by household size for households of 8 or more relative to <8.	*HH.size*	0.447 (0.294, 0.699)
ψ_I	Coefficients modifying infectiousness by viral load and symptom status. Relative to being asymptomatic, *Low.Sym* estimates the effect of shedding low viral load and being symptomatic and *High.Sym* the effect of shedding high viral load and being symptomatic	*Low.Sym*<br>*High.Sym*	2.11 (1.23, 3.66)<br>4.410 (1.82, 8.96)
κ	The rate of exponential decay on the spatial distance kernel	*Dist.rate*	199 (8.28, 206000)
ϑ	The rate of exponential decay on the genetic weight function.	*Gen.rate**	0.000244 (0.000000883, 0.00373)

$\boldsymbol{\varepsilon_g}$	Baseline rate of community exposure by RSV group, per person per day.	*Comm.rsv.a*<br>*Comm.rsv.b*	0.000313 (0.000122, 0.000897)<br>0.000392 (0.000158, 0.00113)
$\boldsymbol{\psi_E}$	Coefficients modifying the rate of community exposure by age group. *Exp.age.2* for 1-4 years and *Exp.age.3* for ≥5 years, relative <1 year	*Exp.age.2*<br>*Exp.age.3*	0.502 (0.208, 1.17)<br>1.58 (0.744, 3.27)
$\boldsymbol{\delta, \beta}$	Parameters for the cluster specific background community function.	*Delta*<br>*Beta*	1.56 (0.541, 4.63)<br>0.194 (0.0834, 0.754)

A3. Model validation

To validate the model, we simulated multiple epidemics and checked to see if the observed epidemic was captured by the range of simulated dynamics. In addition to comparing the time course of cases, we also looked at the total number of cases in an epidemic, the proportion of individuals with multiple onsets and the number of cases in the first and last week of the time period. These values from the data were compared to the range of simulated values to check that key aspects of the epidemic were being reproduced by the simulations.

The results of the model fitting are the posterior parameter distribution and corresponding augmented data for the cluster ids of cases with no genetic information. A simulation based on a set of parameter values will also be based on the corresponding augmented data which will be used to derive a complete set of shedding profiles from the observed data. A single shedding profile is a combination of duration of shedding, viral loads and symptom status, and genetic cluster. The simulation pseudo code per simulation is as follows:

1. Initiate system such that everyone one is susceptible to RSV.
2. At every time step keep track of the following variables:
    a. Exposure status (by RSV cluster)
    b. Shedding status by group
    c. Shedding status by genetic cluster
    d. Infectiousness status (combination of viral load and symptom status)
    e. Infection history (by RSV group)
    f. The background rate of exposure from the community

3. At every time step:

   a. Update the background community function to reflect any new shedding onsets

   b. Calculate the cluster specific rate of exposure, $\lambda_{i,h,c}(t)$, as defined in the main text.

   c. Determine the number of group specific transmission events E_g where

$$E_g = Poisson\left(\sum_{i \in S_{E_g}} P_{E_g,i}\right)$$

S_{E_g} = set of all individuals susceptible to infection event E_g.

$P_{E_g,i}$ = probability of person *i* experiencing event E_g

$$P_{E_g,i} = \sum_{\substack{c\,=\,clusters \\ in\ g}} \left(\left(1 - exp^{-\sum_{c'} \lambda_{i,h,c}(t)}\right) * \left(\frac{\lambda_{i,h,c}(t)}{\sum_{c'} \lambda_{i,h,c}(t)}\right)\right)$$

*Where $\lambda_{i,h,c}(t)$= rate at which person **i** is exposed to infection of cluster type C.*

   d. Given the number of group specific transmission events, determine the cluster id of each through weighted sampling. E.g. if E_g = 4 and ***c*** = {1,2,3} are the cluster ids in the group, the probability of a case being any one if the three clusters is:

$$\left\{\frac{\lambda_{h,1}(t)}{\sum_{c'} \lambda_{h,c}(t)}, \frac{\lambda_{h,2}(t)}{\sum_{c'} \lambda_{h,c}(t)}, \frac{\lambda_{h,3}(t)}{\sum_{c'} \lambda_{h,c}(t)}\right\}, for\ \lambda_{h,1}(t) = \sum_i \lambda_{i,h,c}(t)$$

   e. Determine who experiences each cluster specific transmission event. For a given event, order individuals capable of experiencing the event. For a given person *p* to experience the event, the following inequality has to be satisfied.

$$\sum_{i=1}^{i\leq p-1} P_{E_c,i} < \left(RAND \times \sum_{i \in S_{E_c}} P_{E_c,i} \right) \leq \sum_{i=1}^{i\leq p} P_{E_c,i}$$

Where:

$$P_{E_c,i} = \left(1 - exp^{-\sum_{c'} \lambda_{i,h,c}(t)}\right) * \left(\frac{\lambda_{i,h,c}(t)}{\sum_{c'} \lambda_{i,h,c}(t)}\right)$$

S_{E_c} = all individuals susceptible to infection of cluster type c.

RAND = a random number between (but not including) 0 and 1.

This is illustrated in the figure below.

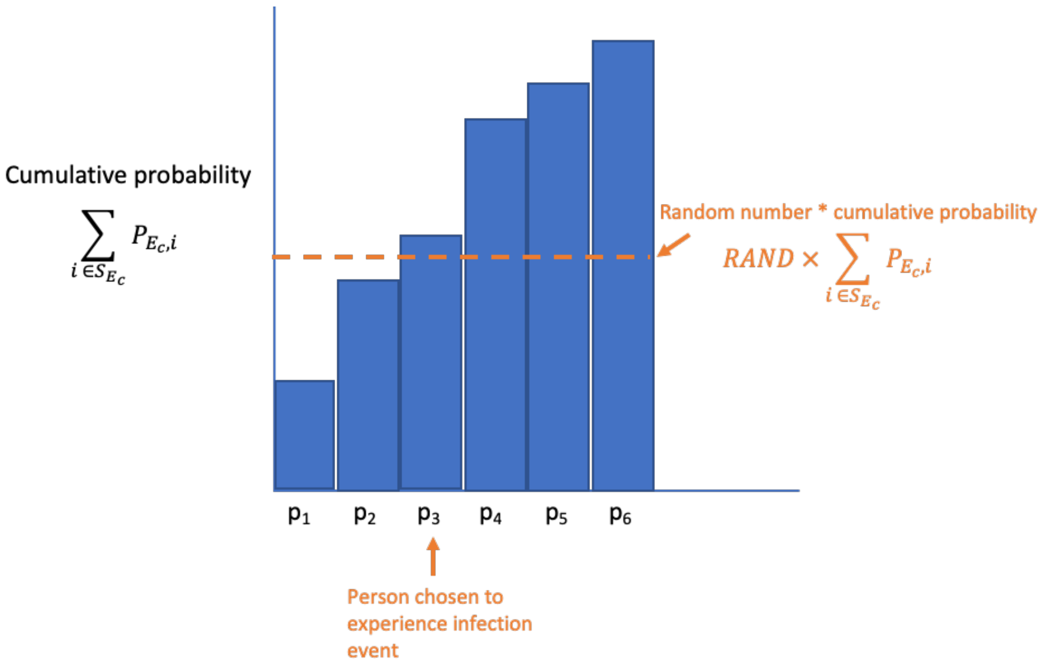

Repeat this until the required number of events

f. For each individual experiencing a transmission event, assign a latency duration and shedding profile by sampling from the relevant empirical

distributions. The empirical latency distribution is the same as was used in estimating the parameters and is homogeneous for every individual. Shedding profiles are derived from the observed data and a combination of duration of shedding, viral loads and symptom status, and genetic cluster. The shedding profiles are grouped by age in the following 4 groups <1,1-5, 5-15 and ≥15 years. Once latency durations and shedding profiles have been assigned, the state variables for each individual are updated accordingly.

To explore how much variation there can be in the simulations from a single parameter set, a set of 12 parameter set samples were used, and for each set, 100 simulations were run, giving a total of 1200 simulations. We then sampled 100 parameter sets and run single simulations from each to explore between-parameter-set variation. The results of the simulations are presented in the form of epidemic curves and summary measures that are used to compare the main features of the outbreak.

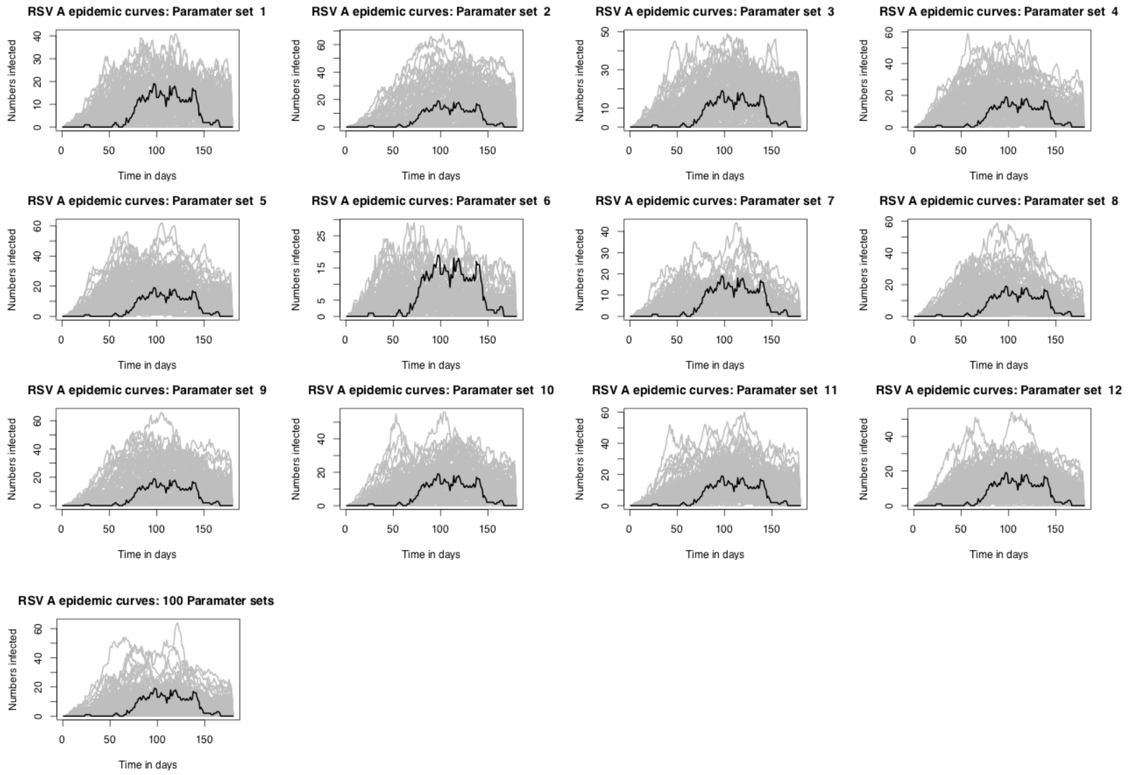

Figure A3. 1: A comparison of simulated and observed data for RSV A.

Each panel shows the results of 100 simulations from a single parameter set. The grey lines show the simulated data while the black lines show the observed data. Time is shown on the x-axis while the y-axis shows the total number of people who are shedding at a given point in time.

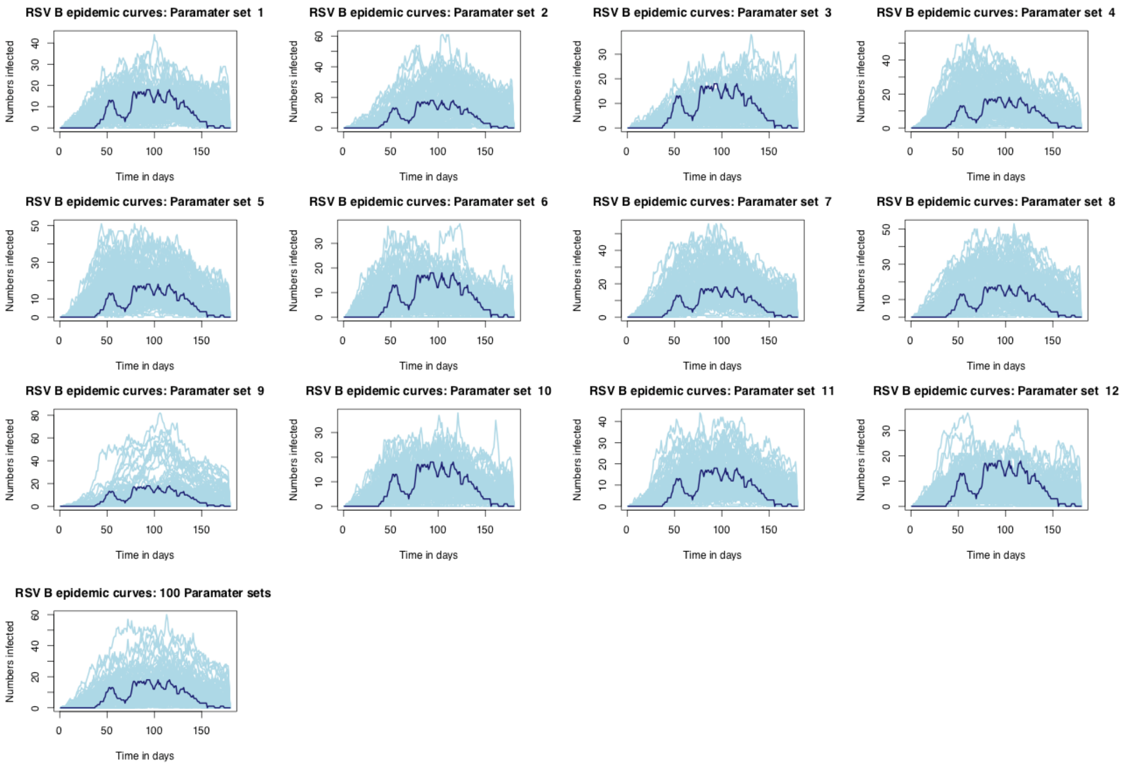

Figure A3. 2: A comparison of simulated and observed data for RSV B.

Each panel shows the results of 100 simulations from a single parameter set. The light blue lines show the simulated data while the dark blue lines show the observed data. Time is shown on the x-axis while the y-axis shows the total number of people who are shedding at a given point in time.

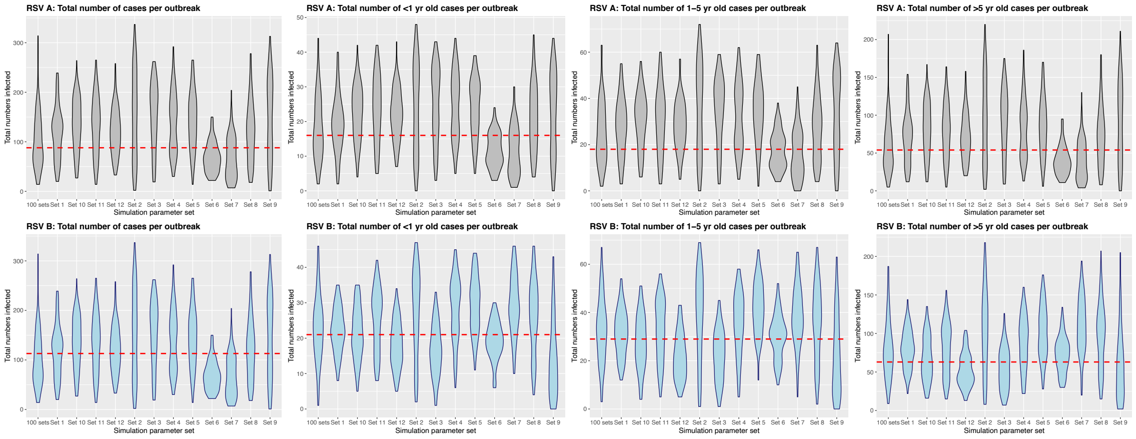

Figure A3. 3: Violin plots showing the distribution of the total number of people infected in the simulations by RSV group and age.

Each panel shows the distribution of the total numbers infected in the simulations run using 12 different parameter sets (violin plots) compared to the total number from the observed data (dashed red line). The y-axis shows the total number and the x-axis is labeled by parameter set used. Top row: RSV A results for all the cases (1st column), cases < 1 year old (2nd column), cases between 1-5 years old (3rd column) and cases > 5 years old (4th column). Bottom row: RSV B results. Violin plots are a combination of box plots and density distributions, the shapes should therefore be interpreted as density plots would while the ranges should be interpreted as the tips of whiskers in a box and whisker plots.

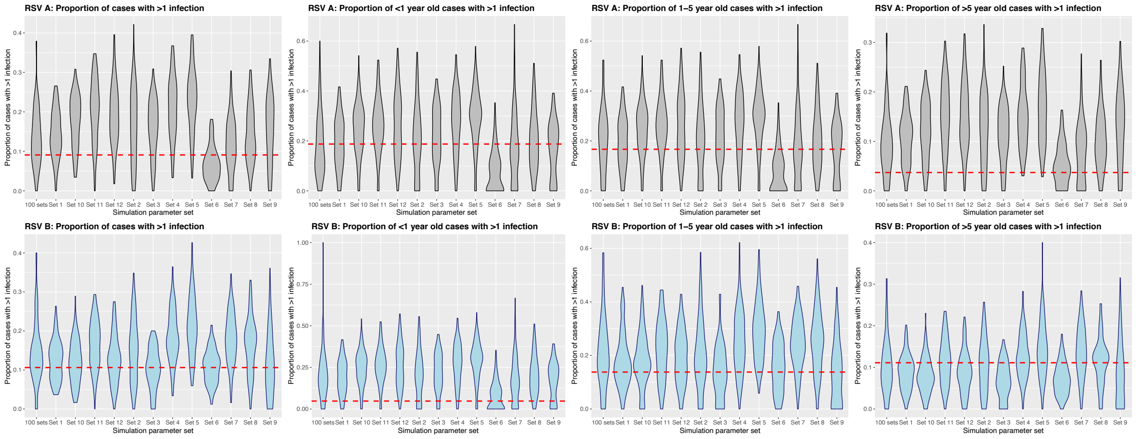

Figure A3. 4: Violin plots showing the distribution of the proportion of cases that had multiple onsets in the simulations by RSV group and age.

Each panel shows the distribution of the proportion of cases that had multiple onsets in the simulations run using 12 different parameter sets (violin plots) compared to the proportion from the observed data (dashed red line). The y-axis shows the proportion and the x-axis is labeled by parameter set used. Top row: RSV A results for all the cases (1st column), cases < 1 year old (2nd column), cases between 1-5 years old (3rd column) and cases > 5 years old (4th column). Bottom row: RSV B results.

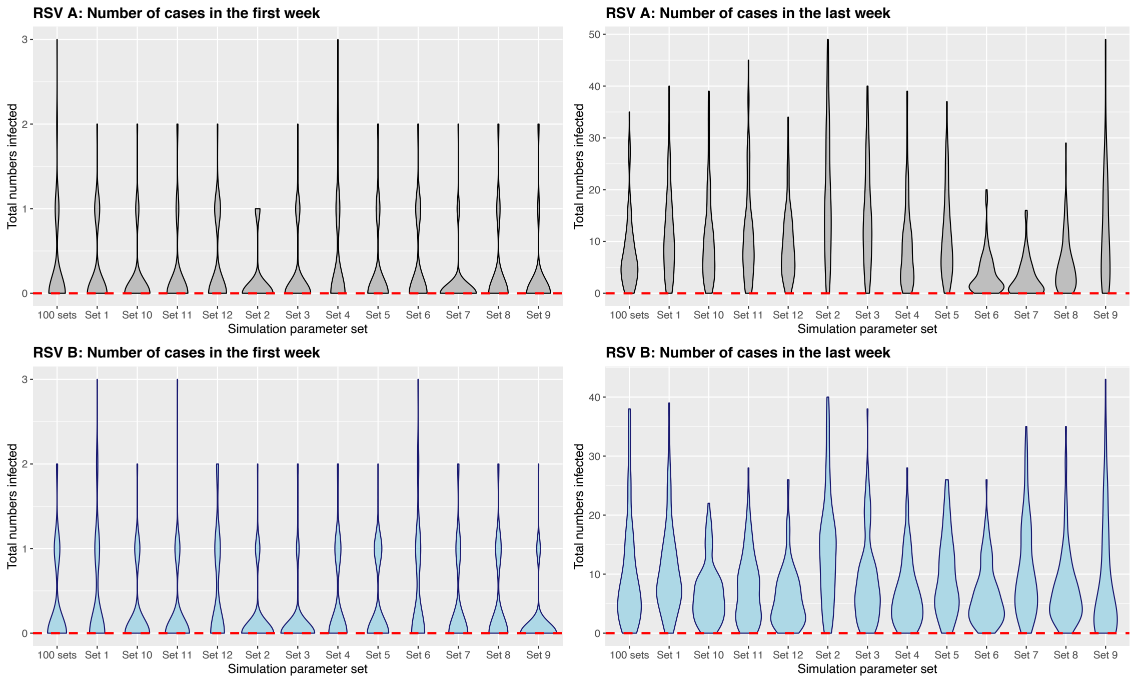

Figure A3. 5: Violin plots showing the distribution of the number of cases in the first (1st column) and last (2nd column) week of the observation/simulation period in the simulations by RSV group.

The y-axis shows the total number of people infected and the x-axis is labeled by parameter set used. The dashed red line shows what was observed in the data, i.e. there were no cases observed in the first and last week of the 180-day observation period.

A4. Further details of the transmission model

The community rate of exposure

Though there are cases from different households in the data, the sample in the study is small relative to the number of households in the community, as such the true number of infectious community contacts is unknown. We therefore split the community rate of exposure into two components: exposure from sampled neighbours, $Sampled\_Neighbour\_Rate_{h,c,j\rightarrow i}(t)$, and exposure from unknown sources represented by a time varying function ***f*_c_(t)**. We assumed that the rate of exposure from a sampled neighbour is dependent on the spatial distance between individuals. $Sampled\_Neighbour\_Rate_{h,c,j\rightarrow i}(t)$ is the cluster specific exposure rate from sampled infectious individual *j* present in a neighbouring household at time *t*, and is given by:

$$Sampled\_Neighbour\_Risk_{h,c,j\rightarrow i}(t) = \psi_{I,inf}\left(Infectivity_{j,h,c}(t)\right) \times K\left(d_{i,j},\kappa\right) \times M_{j,h}(t)$$

The parameter κ is the rate of exponential decay for the spatial distance kernel given by $K\left(d_{i,\mathrm{j}},\kappa\right) = e^{-\kappa * d_{i,\mathrm{j}}}$.

The background community function

We defined a background cluster-specific rate of exposure, $f_c(t)$, which affects susceptible individuals outside their household. This background function allows for introduction of new transmission clusters. The function form for a cluster ***c*** at time ***t*** is given as

$$f_c(t) = \delta + \sum_{\substack{i\ shedding \\ RSV\ cluster\ c}} e^{(t-\tau_{i,c})\beta}$$

Where δ is the basic risk prior to any observed onsets and β is the rate of exponential decay related to the time since onset of a case shedding cluster type ***c***, β is a measure of the rate at which the cluster might disappear from the community and $\tau_{i,c}$ is the onset time of RSV cluster type ***c*** by person ***i***. The parameters δ and β are not cluster or group specific. The sum of the cluster specific curves has to add up to the group specific curve, otherwise using clusters could lead to an over or under representation of the background community exposure rate. To ensure that $\sum f_c(t) = f_g(t)$ we need to normalize the cluster level curves such that their sum adds up to the group level curve. The equation for the normalized function $\hat{f}_c(t)$ is given as:

$$\hat{f}_c(t) = \left(\delta + \sum_{\substack{i\ shedding \\ RSV\ cluster\ c}} e^{(t-\tau_{i,c})\beta} \right) \times \left(\sum_{c \in C'} \left(\delta + \sum_{\substack{i\ shedding \\ RSV\ cluster\ c}} e^{(t-\tau_{i,c})\beta} \right) \right)$$

Where C' is the set of all clusters in a given RSV group.

An example of the shapes of the background community rate of exposure curves is shown in Figure A4. 1 for the 5 clusters in RSV A and Figure A4. 2 for the 7 clusters in RSV B.

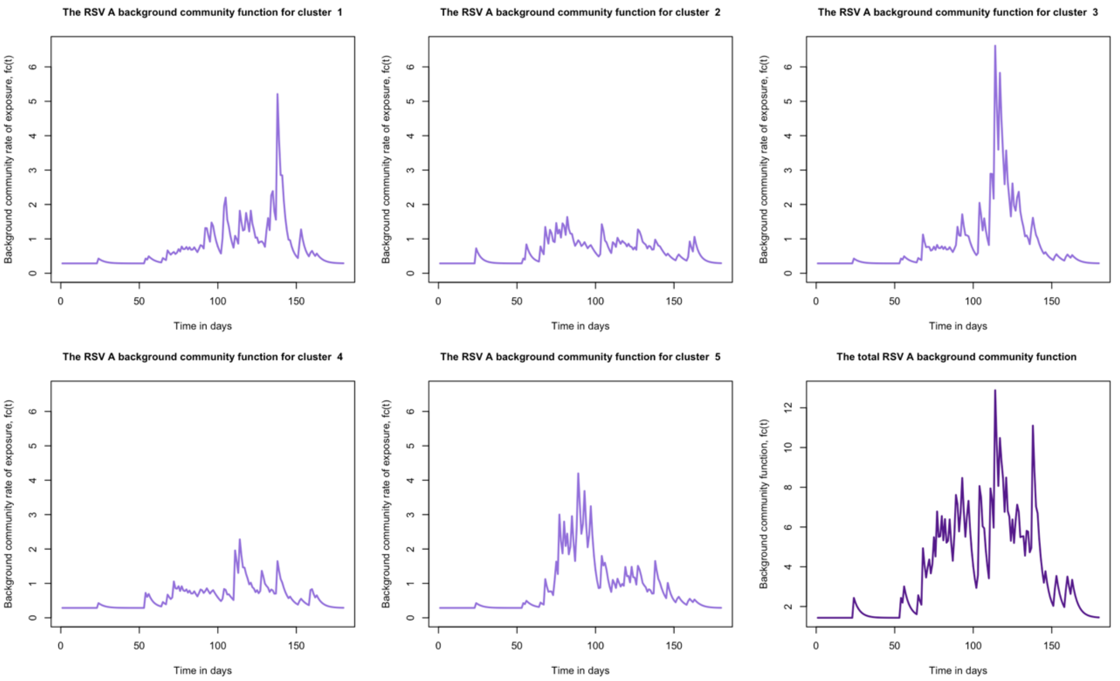

Figure A4. 1: The background cluster-specific rate of exposure curves for RSV A. The normalized $f_C(t)$ curves are shown for the 5 different clusters and the group.

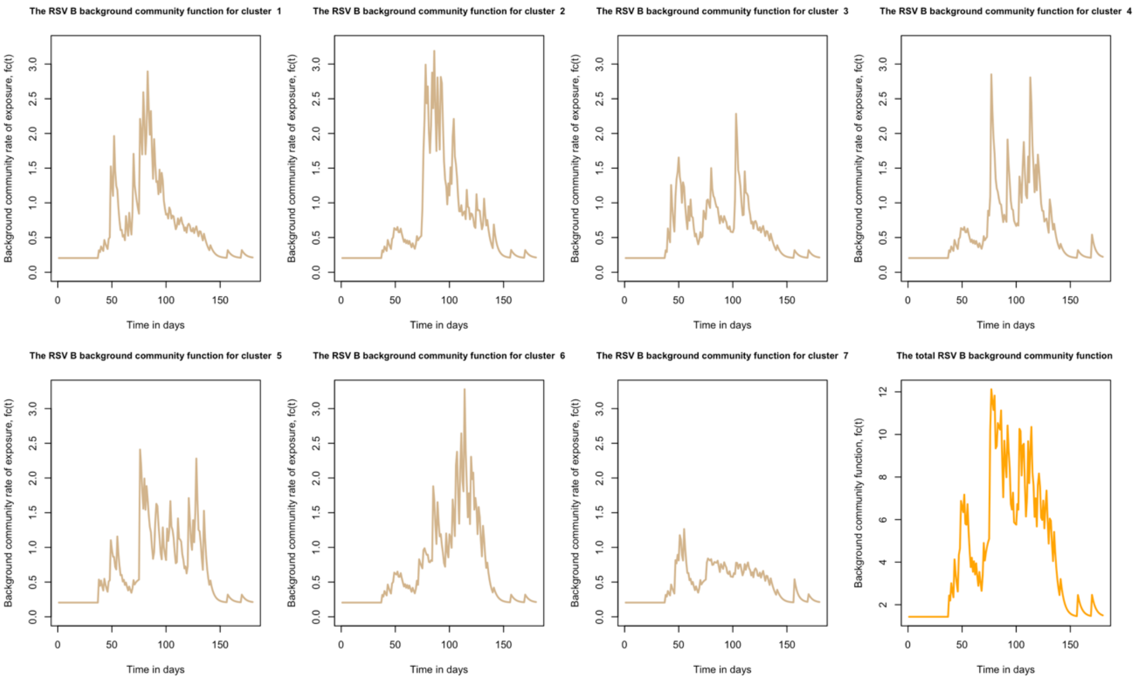

Figure A4. 2: The background cluster-specific rate of exposure curves for RSV B. The normalized $f_C(t)$ curves are shown for the 7 different clusters and the group.

Table A4. 1 lists all the parameters in the model and gives a brief description. Despite identifying the infection pathogen at the cluster level, we do not have any cluster-specific parameters in the model.

Table A4. 1: Model parameters and their descriptions

Parameter (symbol)	Parameter (name)	Description
ϕ_Y	*Prev.hom*, *Prev.het*	Coefficients modifying susceptibility to infection by a particular RSV group depending on infection history. *Prev.hom* estimates the effect of a previous homologous group infection, while *Prev.het* estimates the effect of a previous heterologous group infection
ϕ_X	*Sus.age.2*, *Sus.age.3*, *Sus.age.4*	Coefficients modifying susceptibility to RSV depending on age. *Sus.age.2* estimates the effect being in age group 1-4 years, *Sus.age.3* the effect of group 5-14 and *Sus.age.4* of group ≥15 relative to group <1 year.
ϕ_W	*Curr.het*	Coefficient modifying susceptibility to a particular RSV group based on shedding status of the heterologous group type
η_g	*HH.rsv.a*, *HH.rsv.b*	Baseline rate of within household exposure by RSV group, per person per day.
ψ_H	*HH.size*	Coefficient modifying the amount of within household exposure by household size. *HH.size* estimates the effect

		of being in a large household(>8 inhabitants) relative to a small one
$\boldsymbol{\vartheta}$	*Gen.rate*	For $P_{j\to i} = exp^{-d_{gen}(i,j)*\vartheta}$ the genetic distance kernel giving the genetic weight on probability of transmission, *Gen.rate* is the rate of exponential decay.
$\boldsymbol{\psi_I}$	*Low.Sym*<br>*High.Sym*	Coefficients modifying infectiousness by viral load and symptom status. Relative to being asymptomatic, *Low.Sym* estimates the effect of shedding low viral load and being symptomatic and *High.Sym* the effect of shedding high viral load and being symptomatic
$\boldsymbol{\varepsilon_g}$	*Comm.rsv.a*<br>*Comm.rsv.b*	Baseline rate of community exposure by RSV group, per person per day.
$\boldsymbol{\psi_E}$	*Exp.age.2*<br>*Exp.age.3*	Coefficients modifying the rate of community exposure by age group. *Exp.age.2* estimates the effect being in age group 1-4 years and *Exp.age.3* the effect of group ≥5, relative to the <1-year age group
$\boldsymbol{\kappa}$	*Dist.rate*	The rate of exponential decay for the spatial distance kernel given by $K\left(d_{i,\mathrm{j}}, \kappa\right) = e^{-\kappa * d_{i,\mathrm{j}}}$
$\boldsymbol{\delta}, \boldsymbol{\beta}$	*Delta,*<br>*Beta*	For the cluster specific background community function given by<br>$$f_c(t) = \delta + \sum_{\substack{i\ shedding \\ RSV\ cluster\ c}} e^{(t-\tau_{i,c})\beta}$$

		Delta(δ) is the basic risk and *Beta*(β) is the rate of exponential decay related to the time since onset of a case shedding cluster type ***c***.

Linking the model to data (the likelihood function)

Since the model is focused on the determinants of infection onset process, the data whose likelihood we are interested in is the onset data. Given the model described, the likelihood of an individual's observed cluster ***c*** data is the probability of all the onsets, and days of no onsets where the individual was at risk of infection, i.e. not shedding RSV cluster c. For a particular cluster, this follows a Bernoulli distribution with probability $p_{i,h,c}(u)$.

For ***i*** with no onset of type ***c***:

$$L_{i,c} = \prod_{t=1}^{T}\left[1 - p_{i,h,c}(t)\right]$$

Where *T* is the end of the observation period.

For ***i*** with an onset of type ***c***, the likelihood is given as:

$$L_{i,c} = \left[\left(\prod_{u \in Onsets_{i,h,c}} p_{i,h,c}(u)\right) * \left(\prod_{a \in At_{risk_{i,h,c}}} \left(1 - p_{i,h,c}(a)\right)\right)\right]$$

In this instance, to factor in the genetic data we modify the rate of exposure given in ***Eq 1*** in the main text such that:

$$\begin{aligned} &HH\_Risk_{h,c,j\to i}(t) \\ &\qquad = \eta_g \times \psi_H(Household\_size_i) \times P_{j\to i} \times \psi_{I,inf}\left(Infectivity_{j,h,c}(t)\right) \\ &\qquad \times M_{j,h}(t) \end{aligned}$$

$$Sampled\_Neighbour\_Risk_{h,c,j\to i}(t)$$
$$= P_{j\to i} \times \psi_{I,inf}\big(Infectivity_{j,h,c}(t)\big) \times K\big(d_{i,j},\kappa\big) \times M_{j,h}(t)$$

With this formulation, the genetic components of the model are dependent on the epidemiological in that they are not expressed independently in the likelihood function as is the case with modular approaches such as the kind implemented in the Outbreaker package[1,2]. We introduce $P_{j\to i}$ into the rate of exposure equation as opposed to directly into the likelihood because for a given case, we are not making direct inference on the source of infection or the exact date of exposure: we consider all likely dates and sources given the latency distribution.

The total likelihood is thus given by the product of $L_{i,c}$ over all the genetic clusters and individuals in the data

$$L = \prod_i \left[\prod_c \left[\left(\prod_{u \in Onsets_{i,h,c}} p_{i,h,c}(u) \right) * \left(\prod_{a \in At_{risk_{i,h,c}}} \big(1 - p_{i,h,c}(a)\big) \right) \right] \right]$$

A5. Data pre-processing

Imputing complete shedding, ARI and presence durations.

Given the 3-4 day sampling intervals, complete shedding and ARI durations had to be imputed, and missing viral loads linearly interpolated. For the model, we will assume that all the cases were observed, and ignore the possibility of short duration shedding episodes that could have been missed by the sampling intervals. During the sample-collection visits, if a household member was not present, they were recorded as being ‘away’ on that particular day. As with the shedding information, there was incomplete information on continuous periods of presence or absence from the household which was also imputed.

An RSV A/B shedding episode is defined as a period within which an individual provided PCR positive samples for RSV A/B that were no more than 14 days apart. Using the mid-point method, shedding was assumed to start mid-way between the last negative sample and the first positive sample, and it ended midway between the last positive sample and the first negative sample of an episode. This is illustrated below:

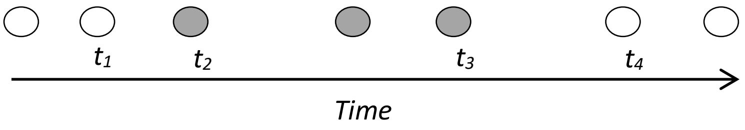

Grey circles are positive samples in a single episode, empty circle are negative. t_1*,* t_2*,* t_3 *and* t_4 *are dates of sample collection.*

For (t_4-t_3) and (t_2-t_1) ≤7 days

$$Duration = \left[t_3 + \left({t_4 - t_3}/_2\right)\right] - \left[t_2 - \left({t_2 - t_1}/_2\right)\right]$$

For (t_4-t_3)>7

$$Duration = \left[t_3 + \left({}^{x}/_{2}\right)\right] - \left[t_2 - \left({}^{t_2 - t_1}/_{2}\right)\right]$$: Right censoring

For (t_2-t_1) >7

$$Duration = \left[t_3 + \left({}^{t_4 - t_3}/_{2}\right)\right] - \left[t_2 + \left({}^{x}/_{2}\right)\right]$$: Left censoring

Where x = mean of sampling intervals for samples in an episode, which was found to be 3.45 days.

Any negative samples (Ct >35 or Ct=0) in between a shedding episode were ignored, i.e. were not treated like true end of shedding

We imputed complete ARI episodes from intervals of recorded ARI. A virus shedding episode that had no day where an ARI was reported was assumed to be asymptomatic. For a virus shedding episode with at least one day of recoded ARI, the duration of symptoms was imputed using the midpoint method described for shedding episodes. This is illustrated below:

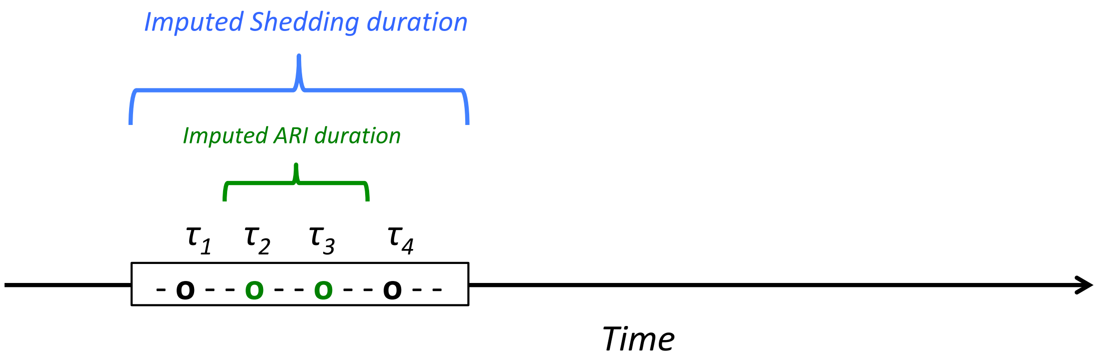

Green open circles are reported ARI symptoms (ARI positive) within the shedding episode and black open circles are confirmed absence of ARI (ARI negative). τ_1, τ_2, τ_3 *and* τ_4 *are days within the shedding episode where information on symptoms was collected.*

In this case, the mean sampling interval for ARI ‘samples’ within an episode was 3.78 days. This was obtained from all ARI episodes not just the ones within shedding episodes

The imputation of continuous periods of presence or absence from the household was done similar to the imputation of shedding durations, however, there was no left or right censoring. Each participant had a set of days of recorded data, these days were either marked as ‘away’ or ‘present’ in the household, e.g. a participant might have data on days {32, 36, 39, 43, 46, 50, 53, 57} with status {away, away, present, away, present, present, away, present}. Since no data is available for this individual before day 32 and after day 57, no imputation is done outside this time window. For the days within the window, imputation is done as illustrated below:

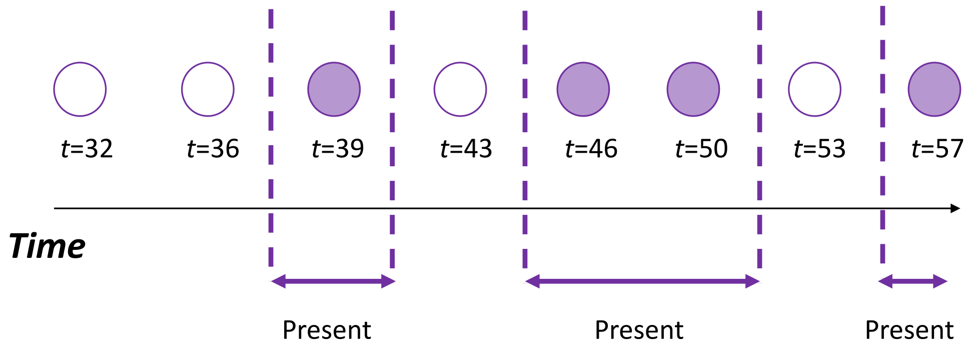

Filled circles are days when the participant was recorded as being present while open ones is when they were away. The present period starts halfway between the last ‘away’ and first ‘present’ and ends halfway between the last ‘present’ and first ‘away’.

In order to include information of the amount of virus shed by an infected person into the transmission model, the Ct value need to be converted to log_{10} RNA copy number which is a more direct measure of viral load. The formula used to convert Ct values to their log_{10} RNA equivalent was $y= -3.308x + 42.9$, where y=Ct values and x=log_{10} RNA copy number[3,4].

Following conversion of the PCR Ct values to viral load, we proceeded to interpolate the viral loads for days in an episode that did not have data. Linear interpolation was used for all the shedding episodes. It was assumed that the starting and ending sample, if data was missing, had a viral load of 2.388 $\log_{10}$ RNA (baseline positive Ct value converted to viral load). For two samples of viral load V_a and V_b at times t_a and t_b, $t_b > t_a$, the gap in between is filled out as follows:

For $t_b - t_a = n$, viral load V_j at time point t_j for j=1…(n-1) is given by

$$V_j = V_a + \frac{j\,(V_b - V_a)}{n}$$

Viral loads lower than 2.388 $\log_{10}$ RNA in between an episode were not included in the interpolation

Imputing missing genetic information

The WGS data was used to rule out transmission events where it was assumed that cases in different genetic clusters are not part of the same transmission cluster. It was also assumed that for cases within the same genetic cluster, the likelihood of a transmission event is weighed according to pairwise genetic distance $d_{gen}(i,j)$. As mentioned in the main text, genetic clusters were established based on a combination of criteria: nucleotide distance cut-off, clustering patterns on the global RSV phylogeny and the inferred date of sequence divergence. As a result of incomplete sequencing of all the positive samples, there are gaps in the genetic data. To fill these in we classified missingness into 3 categories and exploited elements of the study design to fill in the gaps.

- Missing level 1: non-sequenced samples part of an infection episode with ≥1 other sequenced sample. The entire episode was assigned the cluster id of the sequenced sample(s), where there was more than 1 id, the episode was divided accordingly.
- Missing level 2: none of the samples in an episode were sequenced, but the episode is part of a social-temporal cluster with some genetic information. The entire episode was assigned the cluster id of the social-temporal cluster. This assumes that if an episode has a temporal overlap with other cases in the same household, they are likely part of the same infection cluster (household outbreak).
- Missing level 3: none of the samples in an episode were sequenced and there is no genetic information in the social-temporal cluster. The cluster id for the entire episode was treated as augmented data and inferred along with the model parameters.

Within a given RSV group, infection by a particular cluster is assumed to be a mutually exclusive process, an individual can only shed one cluster type at a time. The genetic data available is consensus whole genome sequences as such, only one cluster can be identified from a single sample.

Consider a ***case i*** who had an onset after ***case j***, both of whom have sequences. The genetic distance between case ***i*** and ***j*** is obtained by comparing the first sequence available from case ***i*** and any sequence from ***j*** whose sampling time is closest to the first sequence from ***i***. In the illustration below, this would mean comparing sequence $\boldsymbol{S_{i,1}}$ to $\boldsymbol{S_{j,2}}$ to obtain genetic distance $\boldsymbol{d_{gen}(i,j)}$. The phylogenetic analysis of *Agoti et al* [5] found that long shedding episodes do not have drastically differing genetic sequences (<6 SNPs) as such it should not

make a significant difference whether we compare sequences forward (***S$_{i,1}$*** to ***S$_{j,2}$***) or backward (***S$_{i,1}$*** to ***S$_{j,1}$***) in time.

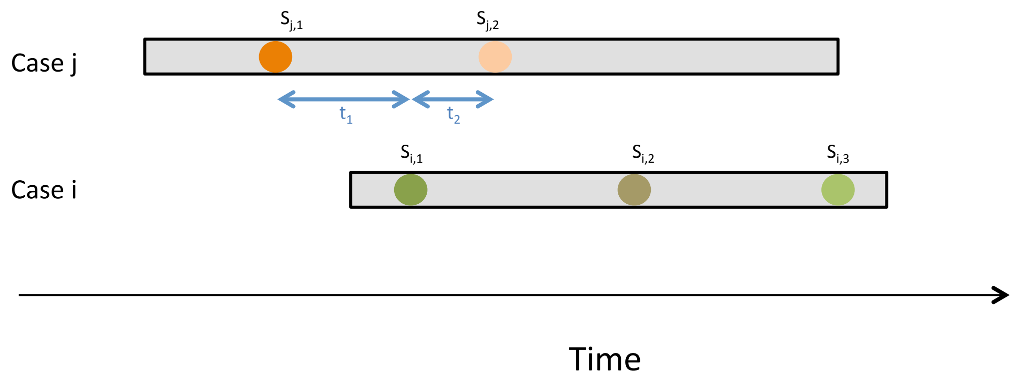

If either one or both of the cases do not have sequences, then $d_{gen}(i,j)$ is randomly selected from the set of all pair-wise genetic distances from the specific genetic cluster. For cases with sequence data, $\boldsymbol{d_{gen}(i,j)}$ is fixed, but for cases where one or both is missing sequences, $\boldsymbol{d_{gen}(i,j)}$ changes every time the likelihood is calculated to reflect uncertainty. In this way only pairs of cases with sequence data contribute definitive genetic information to the parameter inference algorithm while the rest will not. We use nucleotide differences as the distance $\boldsymbol{d_{gen}(i,j)}$. Once we have $\boldsymbol{d_{gen}(i,j)}$, we then use this to obtain a genetic weight for the probability of a transmission event given by $P_{j \to i} = exp^{-d_{gen}(i,j)*\vartheta}$ where ϑ is the rate of exponential decay and is estimated along with other model parameters. This function form results in a negative exponential relationship between the genetic weight and the genetic distance between a pair of cases.

A6. Further details of the adaptive MH-MCMC algorithm

We used Bayesian inference to obtain estimates of the model parameters φ= *{Prev.hom, Prev.het, Sus.age.2, Sus.age.3, Sus.age.4, Curr.het, HH.rsv.a, HH.rsv.b, HH.size, Gen.rate, Low.Sym, High.Sym, Comm.rsv.a, Comm.rsv.b, Exp.age.2,Exp.age.3, Dist.rate, Delta, Beta}* and the augmented data $\boldsymbol{D_A}$ given the observed data $\boldsymbol{D}$. We assume that all the cases were observed but that for some of the cases, there is no information on the cluster id of the shedding episode, as such, the augmented data is the set of all shedding episodes whose cluster id was left unassigned by the imputation process previously described. These include cases that are part of household outbreaks with no genetic information and cases that are part of household outbreaks with more than one possible genetic cluster id. For cases that are part of an outbreak with no genetic information, a single cluster id is inferred for all the cases in the household outbreak.

Bayesian inference results in an updated distribution of the parameter of interest (posterior distribution) given prior assumptions/knowledge of the parameter (prior distribution) and an expression giving the probability of a parameter value given data (likelihood) i.e. $P(\varphi|D, D_A) \propto P(\varphi) \times\ L(\varphi|D, D_A)$. Where there is no exact expression for the posterior distribution, numerical methods are used to find an approximation of the target distribution, adaptive MH-MCMC is a popular first step.

We specified the target distribution as $p(\varphi|D, D_A) = P(D|D_A)L(\varphi|D, D_A)P(\varphi)$; $P(D|D_A)$ = probability of the observed data given the augmented data; $L(\varphi|D, D_A)$ = the likelihood of the parameters given the observed and augmented data; $P(\varphi)$ = the prior probability of the parameters. The augmented and observed data are independent and we have no

information to inform what the missing cluster ids could be, making every combination of D and D_A equally likely. Consequently, we did not include $\mathrm{P(D|A)}$ when calculating the posterior probability. We used weakly informative priors in the form of a normal distribution with mean 0 and a standard deviation of ~3 for the log of parameters. We initiated 3 chains and set the algorithm to start adapting the proposal distribution based on accepted parameters after 10000, 15000 and 10000 iterations respectively. Burn-in was assessed visually after which the results of the three concurrent chains were combined to infer the posterior distribution. The three chains were run for 250,000 iterations each.

Choice of proposal distributions for the parameters

For the parameter set φ we used a multivariate normal distribution as the proposal distribution. For iteration *n* in the chain a new set φ^* will be proposed such that $\varphi^* \sim Normal(\varphi^{n-1}|\Sigma)$. The choice of the variance-covariance matrix Σ will determine the size of the space that is explored and how fast the MCMC chain converges. After a certain number of iterations, Σ was modified to ensure proper mixing. The modification was automated through an adaptive random walk MH-MCMC algorithm. There are several adaptation algorithms [6], we chose one that learns from the empirical distribution of values up to the (n-1)th iteration to modify the Σ at iteration n. For samples $\{\varphi_1, \varphi_2, \varphi_3, ... \varphi_{n-1}\}$ in the MCMC chain so far, at iteration n the proposal density g(.) is given by

$$g_n(.) = (1-\varepsilon)N(\varphi^{n-1}|2.38^2\Sigma_{n-1}/d)) + \ \varepsilon N(\varphi^{n-1}|0.1^2\Sigma_0/d)$$

Where:

$\varepsilon =$ A small positive constant, chosen to be 0.05 as in [6].

Σ_{n-1} = The empirical variance-covariance matrix derived from samples $\{\varphi_1, \varphi_2, \varphi_3, \dots \varphi_{n-1}\}$

d = The dimension of the parameter set

Σ_0 = The initial guess of the parameter variance-covariance matrix. This is usually a diagonal matrix of variances.

This notation means for a fraction of the time $(1-\varepsilon)$, the proposal distribution will be $N(\varphi^{n-1}|2.38^2\Sigma_{n-1}/d))$ and the rest of the time it will be $N(\varphi^{n-1}|0.1^2\Sigma_0/d)$. Prior to adaptation beginning at iteration *n*, the proposal distribution at iteration *k* is given by $g_k(.) = N(\varphi^{k-1}|0.1^2\Sigma_0/d)$

Pseudo algorithm for our implementation of MH-MCMC

For each MCMC chain

1. Set initial values for the parameters and assign cluster ids at random for the outbreaks with no sequence information (uninformed outbreaks).
2. For every iteration n
    a. Update parameter values
        i. Propose a new set of parameters by sampling from the proposal distribution: $\varphi^* \sim Normal(\varphi^{n-1}|\Sigma)$
        ii. Calculate the acceptance probability $\rho(\varphi^{n-1}, \varphi^*) = \min\left\{1, \frac{p(\varphi^*|D, D_A^{n-1})}{p(\varphi^{n-1}|D, D_A^{n-1})}\right\}$
        iii. If $\rho(\varphi^{n-1}, \varphi^*) > r \sim Uniform(0,1)$ update $\varphi^n = \varphi^*$ otherwise $\varphi^n = \varphi^{n-1}$

b. Update cluster id for a single uniformed outbreak

i. Randomly select an uniformed outbreak from the set of uninformed outbreaks, all with the same probability of being selected.

ii. Given the present cluster id for the chosen outbreak C_r, randomly select a new cluster id from the set of all possible clusters excluding C_r.

iii. With C_s as the proposed cluster id, the proposed change to the augmented data is accepted with probability

$$\rho'(D_A^{n-1}, D_A^*) = \min\left\{1, \frac{p(\varphi^n|D, D_A^*)}{p(\varphi^n|D, D_A^{n-1})}\frac{|C_r|}{|C_s|+1}\right\}$$

Where $|C_r|$ is the number of household outbreaks in C_r in the present permutation of the augmented data D_A^{n-1} and $|C_s|$ is the number of household outbreaks in C_s.

iv. If $\rho'(D_A^{n-1}, D_A^*) > r' \sim Uniform(0,1)$ update D_A^n, D_A^* otherwise D_A^n, D_A^{n-1}

The correction factor $\frac{|C_r|}{|C_s|+1}$ is introduced into the acceptance ratio for a proposed change in cluster id because the proposal distributions are not symmetric. For an update of cluster id from C_S to C_r, the proposed change is uniformly distributed over the set of all household outbreaks/cases in cluster C_S that are part of the augmented dataset. Conversely the reverse move of a change of cluster id from C_r to C_S is uniformly distributed over the set of all household outbreaks/cases in cluster C_r that are part of the augmented dataset. As such,

the proposal distributions are dependent on the number of uniformed household outbreaks in each cluster.

A7. Further details of the HPTS

Establishing the highest probability transmission source (HPTS)

Per case, we identified the transmission source that had the highest likelihood given the data and a parameter set φ^* sampled from the joint parameter posterior distribution (highest probability transmission source: HPTS). Consider a case ***i*** ,with onset date T_i^O. Given our assumption of a maximum latency duration of 5 days, we define a time window where potential infection could have occurred. For each day in the time window, potential sources of infection are $\{\Omega_i^1, \Omega_i^2 \dots \Omega_i^n\}$. An infection source is assigned if it gives the highest value of ***i***'s likelihood defined as “ the likelihood of ***i***'s onset date, infection date and infection source given sample parameter set φ^*.

We modified the likelihood to establish the most likely infection source (HPTS) for every case. For a given case ***i*** infected with RSV cluster ***c*** within group ***g***, there are three possible sources of infection (Ω_i), either a sampled housemate, a sampled neighbour or an unknown community source. The total rate of exposure is given as:

$$\lambda_{i,h,c}(t) = S_{i,g}(t)\left[M_{i,h}(t)\sum_{j\neq i} HH_{Rate_{h,c,j\to i}}(t) + \ Comm\_Rate_{i,c}(t)\right] \qquad \dots (Eq\ A4.1)$$

Where (as in the main text):

$S_{i,g}(t)$ is the factor modifying exposure by recent group specific infection history, age and group specific shedding status at time ***t***

$Comm\_Rate_{i,c}(t)$ is the cluster specific community (external to the household) exposure rate.

The probability of exposure is = *prob(any exposure event) * prob(exposure to cluster c)*

$$\alpha_{i,h,c}(t) = \left(1 - exp^{-\sum_{c'} \lambda_{i,h,c}(t)}\right) * \left(\frac{\lambda_{i,h,c}(t)}{\sum_{c'} \lambda_{i,h,c}(t)}\right) \qquad \dots (Eq\ A4.2)$$

For a given source of infection Ω_i in the same household as ***i***, the rate of exposure is given by:

$$\lambda_{\Omega_i \to i,h,c}(t) = S_{i,g}(t)\big[M_{i,h}(t) \times P_{\Omega_i \to i} \times \eta_g \times \psi_H(Household\_size_i) \times \psi_{I,inf}\big(Infectivity_{\Omega_i,h,c}(t)\big) \times M_{\Omega_i,h}(t)\big]$$

For Ω_i not in the same household as ***i*** but among the sampled individuals, the rate of exposure is given by:

$$\lambda_{\Omega_i \to i,h,c}(t) = S_{i,g}(t)\left[\varepsilon_g \times \psi_{E,age}\left(Age_{group_{E,i}}\right) \times M_{i,h}(t) \times P_{\Omega_i \to i} \times \psi_{I,inf}\left(Infectivity_{\Omega_i,h,c}(t)\right) \times K\left(d_{i,\Omega_i}, \kappa\right) \times M_{\Omega_i,h}(t)\right]$$

For Ω_i an unknown source external to the household, the rate of exposure is given by:

$$\lambda_{\Omega_i \to i,h,c}(t) = S_{i,g}(t)\left[\varepsilon_g \times \psi_{E,age}\left(Age_{group_{E,i}}\right) \times f_c(t)\right]$$

The probability of transmission from a single source Ω_i at time *t* thus becomes:

$$Pr_{\Omega_i \to i,h,c}(t) = \frac{\lambda_{\Omega_i \to i,h,c}(t)}{\lambda_{i,h,c}(t)} \qquad \dots (Eq\ A4.3)$$

The likelihood function

The probability given in $(Eq\ A4.1)$ is calculated for a time point *t* = exposure time of individual *i*, t_i^E. This is not observed in the data, however, given our assumption on the latency duration, we can define a 6-day window of possibility. If case *i* had a shedding onset at time T_i^O, then the window for transmission is from day $\left(T_i^O - 5\right)$ to $\left(T_i^O - 0\right)$. For each day in the window, potential sources are identified based on shedding status and for each combination of infection source Ω_i and exposure date t_i^E, the likelihood is calculated using the formula below:

$$L(\varphi \left| \{T_i^o, t_i^E, \Omega_i\}\right) = \alpha_{i,h,c}(t) * \left(\prod_{t_i \neq t_i^E} \left(1 - \alpha_{i,h,c}(t)\right) \right) * \left(\theta_l(T_i^o - t_i^E)\right) * \left(\frac{\lambda_{\Omega_i \to i,h,c}(t_i^E)}{\lambda_{i,h,c}(t_i^E)} \right)$$

The first part of the product is the probability of infection with cluster ***c*** at time t_i^E, the second part is the probability of escaping infection at any time $t_i \neq t_i^E$, the third is the probability of a latency duration of length $(T_i^o - t_i^E)$ and the last term is the probability of transmission from source Ω_i to *i*.

Given the likelihood, the highest-probability-source is chosen as the infection source that gives the highest value of the likelihood.

A8. References

1. Campbell, F. *et al.* outbreaker2 : a modular platform for outbreak reconstruction. **19**, (2018).
2. Jombart, T. *et al.* Bayesian Reconstruction of Disease Outbreaks by Combining Epidemiologic and Genomic Data. *PLoS Comput. Biol.* **10**, e1003457 (2014).
3. Nolan, T., Hands, R. E. & Bustin, S. A. Quantification of mRNA using real-time RT-PCR. *Nat. Protoc.* **1**, 1559–1582 (2006).
4. Wathuo, M., Medley, G. F., Nokes, D. J. & Munywoki, P. K. Quantification and determinants of the amount of respiratory syncytial virus (RSV) shed using real time PCR data from a longitudinal household study. *Wellcome Open Res.* **1**, 27 (2017).
5. Agoti, C. N. *et al.* Genomic analysis of respiratory syncytial virus infections in households and utility in inferring who infects the infant. *Sci. Rep.* **9**, 10076 (2019).
6. Roberts, G. O. & Rosenthal, J. S. Examples of Adaptive MCMC. *J. Comput. Graph. Stat.* **18**, 349–367 (2009).
